# Chinese Herbal Medicine for Type 2 Diabetes Mellitus With Nonalcoholic Fatty Liver Disease: A Systematic Review and Meta-Analysis

**DOI:** 10.3389/fphar.2022.863839

**Published:** 2022-06-27

**Authors:** Sihan Peng, Lu Liu, Ziyan Xie, Xiyu Zhang, Chunguang Xie, Sha Ye, Xiangeng Zhang, Xiaoli Liang, Hongyan Wang, Ya Liu

**Affiliations:** ^1^ Hospital of Chengdu University of Traditional Chinese Medicine, Chengdu, China; ^2^ Chengdu University of Traditional Chinese Medicine, Chengdu, China; ^3^ No. 1 Orthopedics Hospital of Chengdu, Chengdu, China; ^4^ Sichuan Nursing Vocational College, Chengdu, China

**Keywords:** type 2 diabetes mellitus(T2DM), nonalcoholic fatty liver disease(NAFLD), Chinese herbal medicine (CHM), meta-analysis, systematic review

## Abstract

**Objectives:** To evaluate the efficacy and safety of Chinese herbal medicine (CHM) for type 2 diabetes mellitus (T2DM) with nonalcoholic fatty liver disease (NAFLD) with current evidence.

**Methods:** This study was registered in PROSPERO as CRD42021271488. A literature search was conducted in eight electronic databases from inception to December 2021. The primary outcomes were lipid indices and liver functions, including triglyceride (TG), total cholesterol (TC), low-density lipoprotein cholesterol (LDL-C), high-density lipoprotein cholesterol (HDL-C), alanine transaminase (ALT), and aspartate transaminase (AST). Review Manager 5.2 and Stata v14.0 were applied for analysis.

**Results:** The research enrolled 18 RCTs with 1,463 participants. Results showed CHM combined with western medicine (WM) was more effective than WM alone in TG (weighted mean differences (WMD) = −0.35.95% confidence interval (CI) [−0.51, −0.19], *p* < 0.0001), TC (WMD = −0.58.95%CI [−0.80, −0.36], *p* < 0.00001), LDL-C (WMD = −0.37, 95%CI [−0.47, −0.26], *p* < 0.00001), HDL-C (WMD = 0.20, 95%CI [0.10, 0.29], *p* < 0.0001), ALT (WMD = −4.99, 95%CI [−6.64, −3.33], *p* < 0.00001), AST (WMD = −4.76, 95%CI [−6.35, −3.16], *p* < 0.00001), homeostatic model assessment of insulin resistance (WMD = −1.01, 95%CI [−1.22, −0.79], *p* < 0.00001), fasting blood glucose (WMD = −0.87, 95%CI [−1.13, −0.61], *p* < 0.00001), 2-h postprandial glucose (WMD = −1.45.95%CI [−2.00, −0.91], *p* < 0.00001), body mass index (WMD = −0.73.95%CI [−1.35, −0.12], *p* = 0.02), and overall effective rate (risk ratio (RR) = 1.37.95%CI [1.29, 1.46], *p* < 0.00001).

**Conclusion:** The CHM in combination with WM seems to be more beneficial in T2DM with NAFLD patients in improving lipid and glucose metabolism, liver function, and insulin resistance as well as improving overall efficiency and reducing body weight. Given the poor quality of reports from these studies and uncertain evidence, these findings should be interpreted cautiously.

**Systematic Review Registration:** https://www.crd.york.ac.uk/prospero/display_record.php?ID=CRD42021271488, identifier CRD42021271488.

## 1 Introduction

Nonalcoholic fatty liver disease (NAFLD), also known as metabolism-associated fatty liver disease (MAFLD), represents a clinical syndrome characterized by steatosis and accumulation of fat in liver parenchymal cells (Eslam et al., 2020). NAFLD includes simple fatty liver, nonalcoholic steatohepatitis (NASH), and cirrhosis (Younossi et al., 2018). The prevalence of NAFLD is as high as 75% in population diagnosed with type 2 diabetes mellitus (T2DM) (Hua et al., 2014; Younossi et al., 2016). T2DM and NAFLD can accelerate disease progression in a reciprocal manner, thereby becoming an important social public health burden (Loria et al., 2013; Mantovani et al., 2018). NAFLD could increase glycemic excursions, making it more challenging to glycemic control glycemic (Bril and Cusi, 2017), as a result significantly increase the risk of macrovascular and microangiopathy (Hazlehurst et al., 2016; Lomonaco et al., 2016). T2DM, on the other hand, exacerbates the risk of liver fibrosis and cancer in NAFLD (Targher et al., 2021). The coexistence of the two pathologies leads to a significant increase in the risk of aggravated metabolic disorders and cardiovascular disease, culminating in death related to liver disease (Portillo-Sanchez et al., 2015). Most T2DM patients with NAFLD experience “late detection, late treatment, and difficulty in recovery” because of the lack of typical or serious clinical symptoms or signs in the early stages, thereby impacting the physical and mental health and quality of life (Hu et al., 2017).

Obesity and insulin resistance (IR) are major pathogenic drivers in NAFLD and T2DM (Buzzetti et al., 2016; Wei et al., 2021), and these two pathological conditions usually coexist (Tilg et al., 2017; Targher et al., 2018). Due to the prevalence of T2DM with NAFLD and the many potential health risks associated with their coexistence, active and effective prevention measures should be employed to protect this population (Wong et al., 2018; Lee et al., 2020). At present, there is no effective pharmacotherapy for NAFLD approved by the international authorities, regardless of T2DM status. (European Association for the Study of the Liver, 2016). Lifestyle modifications are key to the clinical management of NAFLD across the disease spectrum (Nguyen and George, 2015). Both T2DM and NAFLD promote deterioration of each other physiologically and pathologically, and there are many restrictions in treatment, thereby precluding an effective interventional strategy. Therefore, alternative therapy is urgently needed.

Traditional Chinese medicine (TCM) is one of the major complementary and alternative medicine systems that have been developed in China for thousands of years, including Chinese herbal medicine (CHM), acupuncture, massage, and other therapies. In addition to China, some Asian countries also have high acceptance of CHM. CHM is a traditional botanical, animal, and mineral medicine used in China, of which botanical is the most common. CHM, based on the principles of syndrome differentiation and holistic treatment, has a long history in the treatment of diabetes and its complications, which can provide individualized treatment for patients. Existing studies (Chen et al., 2016; Shi et al., 2019; Wu and Wei, 2019) have described CHM as effective in the treatment of T2DM with NAFLD and is different from the forced hypoglycemic and lipid-lowering effects of Western medicine (WM), thereby providing a therapeutic advantage of “all-round, multi-faceted, and multi-target.” More recent traditional Chinese medicine (TCM) scholars have studied CHM purely based on theoretical considerations, clinical applications, and scientific experiments and demonstrated that CHM can effectively regulate glucose and lipid metabolism, improve insulin resistance and hemorheology (Gao et al., 2020), repair liver histopathological injury (Chen C. et al., 2021), inhibit oxidative stress (Zhang L. L. et al., 2019), and delay the progression of T2DM with NAFLD. Nevertheless, to date, there is no systematic review or meta-analysis to evaluate the efficacy and safety of CHM. In this study, we addressed the efficacy and safety of CHM in relation to the management of T2DM with NAFLD using an evidence-based approach, with the aim of providing scientific references for improving the therapeutic strategy.

## 2 Methods

This study was conducted in accordance with the Cochrane Handbook on Systematic Review of Interventions, the guidelines of Preferred Reporting Items for Systematic Review and Meta-Analysis Protocols (PRISMA-P) 2015 (Moher et al., 2015) (Supplementary File S1). Additionally, the review was registered at PROSPERO (CRD42021271488).

### 2.1 Search Strategies

We performed a comprehensive search of eight electronic databases from inception to December 2021, including PubMed, Cochrane Library, Embase, Web of Science, China National Knowledge Infrastructure (CNKI), Wanfang Database, China Biomedical Medicine database (CBM), and the VIP information resource integration service platform (cqvip). In addition, the Chinese Clinical Trial Registry (CHiCTR) (http://www.chictr.org.cn/index.aspx) and ClinicalTrials.gov were also searched for randomized controlled trials (RCTs) that were either ongoing or completed but unpublished. We included all RCTs that examined the efficacy of CHM in the management of T2DM with NAFLD. Search terms included were as follows: “Traditional Chinese Medicine,” “Traditional Tongue Diagnosis,” “Zhong Yi Xue,” “Chung I Hsueh,” “Diabetes Mellitus, Type 2,” “Diabetes Mellitus, Noninsulin-Dependent,” “Stable Diabetes Mellitus,” “Diabetes Mellitus, Type II,” and “Maturity-Onset Diabetes,” etc. Comprehensive search strategies for the databases are shown in the Supplementary Files (Supplementary Table S1). No restrictions were applied on language.

### 2.2 Inclusion and Exclusion Criteria

All RCTs evaluating the effects of CHM on T2DM with NAFLD were included in the meta-analysis. The inclusion criteria were as follows:

1) *Study design*: RCTs; 2) *Participants*: patients with a definite diagnosis of T2DM with NAFLD and no limitations relating to gender, nationality, ethnicity, and education level. 3) *Interventions*: patients in the intervention group should receive CHM (including decoction, pills, and granules, regardless of duration) plus WM, and the control group should be treated with WM the same as the intervention group; 4) *Outcomes*: the primary outcomes of the study were triglyceride (TG), total cholesterol (TC), low-density lipoprotein cholesterol (LDL-C), high-density lipoprotein cholesterol (HDL-C), alanine transaminase (ALT), and aspartate transaminase (AST). The secondary outcomes included were homeostatic model assessment of insulin resistance (HOMA-IR), fasting blood glucose (FBG), 2-h postprandial glucose (2hPG), body mass index (BMI), overall effective rate, and adverse effects. Studies that met any of the following criteria were excluded: 1) non-RCTs, such as retrospective studies, animal experiments, case reports, reviews, and conference abstracts. 2) Patients received other TCM interventions, including acupuncture, massage, or moxibustion. 3) Studies that lacked sufficient details on outcomes.

### 2.3 Study Selection and Data Extraction

Three investigators (LL, ZX, and X-YZ) searched and screened for appropriate studies according to the predefined inclusion and exclusion criteria. In the case of discrepancies, the final decision was made through consensus agreement. To manage literature, Endnote V.X9 software was used. Two reviewers (HW and XL) independently extracted relevant data from the eligible studies using standardized extraction forms, including: the first author, year of publication, country, sample size, average age, gender, duration of disease, interventions, details of CHM (name of the prescription and composition), adverse events, and outcomes. The extracted data were cross-checked by HW and XL, and a third reviewer (SY) was available to resolve any conflicts.

### 2.4 Risk of Bias Assessment

Two reviewers (CX and X-GZ) independently assessed the risk of bias according to the Cochrane Collaboration’s Risk of Bias tool (Higgins et al., 2019), which included the following criteria: random sequence generation, allocation concealment, incomplete data, blinding, selective reporting, and other bias. The results were judged as ‘low,’ ‘high,’ or ‘unclear,’ and any disagreements were resolved by the third investigator (SY).

### 2.5 Data Synthesis and Statistical Analysis

Review Manager 5.2 was applied to analyze and assess the effect of CHM on T2DM with NAFLD patients from the aspects of lipid indices, liver functions, insulin, glycemic indices, and so on. For dichotomous data, a risk ratio (RR) with a 95% confidence interval (CI) was used to measure the results. Continuous variables, such as TG, TC, ALT, AST, FBG, and BMI, were evaluated by weighted mean differences (WMDs) and 95%CI. The heterogeneity of data was investigated by the X^2^ test and I^2^ test. A fixed effects model was applied if there was homogeneity (*p* > 0.05, I^2^<50%) (Higgins et al., 2003); otherwise, the random effects model was used. A *p*-value of less than 0.05 was considered statistically significant. To explore the potential sources of heterogeneity, the factors that contributed to heterogeneity were analyzed through subgroup analysis. In addition, publication bias was assessed by funnel plots and investigated statistically by Egger’s test with Stata v14.0.

### 2.6 Sensitivity Analysis

To assess the robustness and reliability of the combined results in meta-analysis, we used sensitivity analysis as an important method. Sensitivity analysis was conducted by excluding individual studies in-turn and re-performing the meta-analysis of the remaining studies. We evaluated whether the results obtained were significantly different from those before the exclusion to ensure the robustness of the results.

## 3 Results

### 3.1 Literature Selection

**FIGURE 1:**
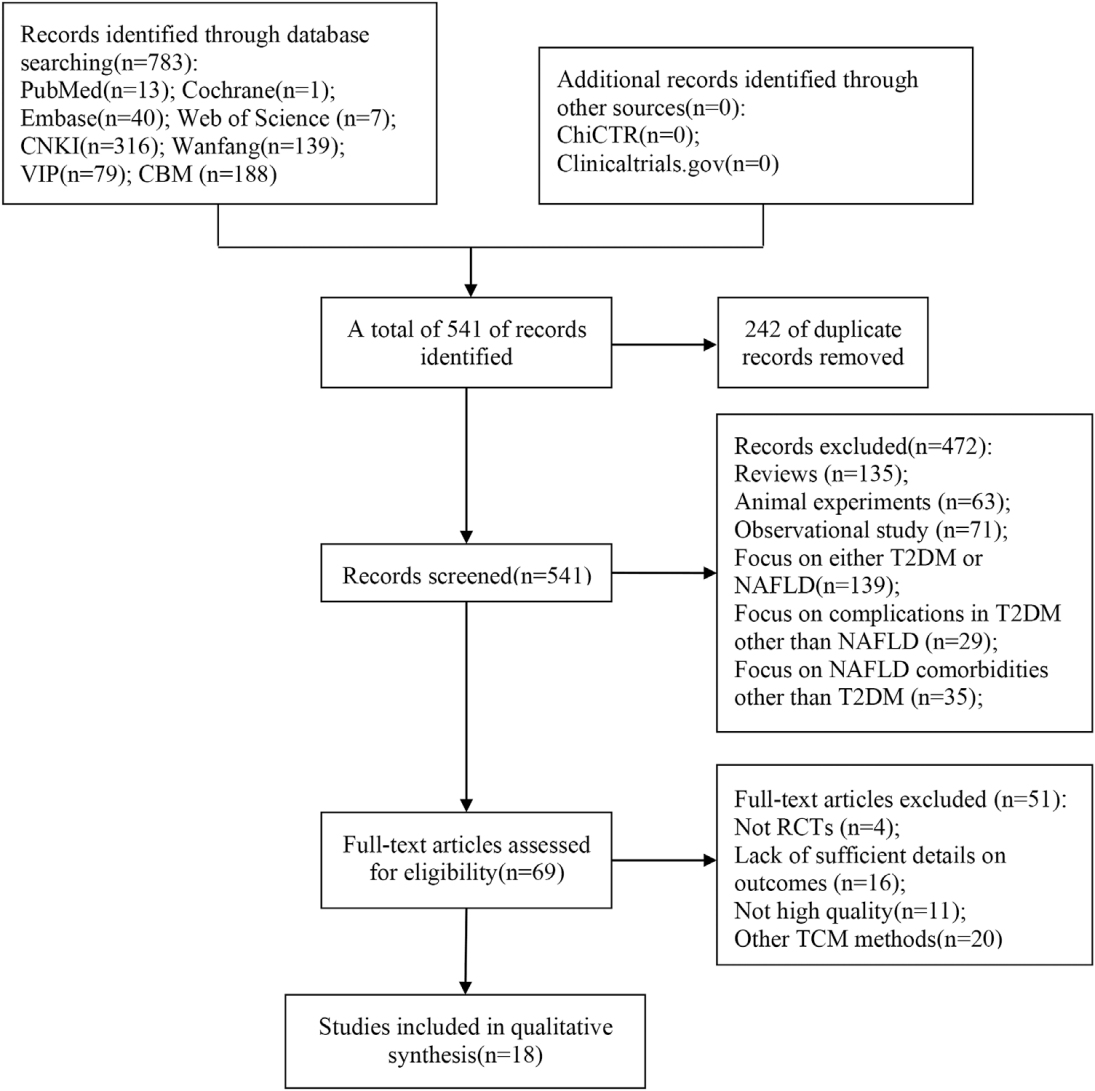
Flow diagram of studies selection process.

Through our search strategy, 783 relevant articles were initially evaluated. After removing duplicates, the remaining 541 articles were screened by title and abstract, and 69 articles required further screening after excluding articles that did not meet the inclusion criteria, such as reviews and animal experiments. After careful full-text reading of the 69 articles, 51 articles were excluded for the following reasons: not RCTs (*n* = 4), other TCM methods (*n* = 20), lack of sufficient details on outcomes (*n* = 16), and lack of high quality (*n* = 11). Finally, 18 articles (Wang, 2010; Kang et al., 2016; Zheng et al., 2016; Xia, 2017; Chen, 2018; Guan et al., 2018; Wang, 2018; Wang et al., 2018; Li, 2019; Liu, 2019; Wang, 2019; Zou, 2019; Li et al., 2020; Liu, 2020; Wang, 2020; Chen M. L. et al., 2021; Fan et al., 2021; Pu et al., 2021) were included in this meta-analysis. The study selection process is shown in Figure 1.

### 3.2 Study Characteristics

A total of 18 studies involving 1,463 patients were enrolled, 733 in treatment and 730 in control groups. All studies were RCTs conducted in China between 2010 and 2021. In these trials, the intervention of treatment groups was to add CHM to control groups, while there were two kinds of interventions in control groups: 7 studies (Wang, 2010; Kang et al., 2016; Zheng et al., 2016; Chen, 2018; Wang et al., 2018; Li, 2019; Li et al., 2020) used hypoglycemic drug, while 11 studies (Xia, 2017; Guan et al., 2018; Wang, 2018; Liu , 2019; Wang, 2019; Zou, 2019; Liu, 2020; Wang, 2020; Chen M. L. et al., 2021; Fan et al., 2021; Pu et al., 2021) used the combination of hypoglycemic drug and hypolipidemic drug. In 18 RCTs, CHM in the treatment group was taken in the form of decoction in 14 studies (Wang, 2010; Xia, 2017; Chen, 2018; Guan et al., 2018; Wang, 2018; Wang et al., 2018; Li, 2019; Liu, 2019; Wang, 2019; Zou, 2019; Liu, 2020; Wang, 2020; Fan et al., 2021; Pu et al., 2021), granules in 2 studies (Kang et al., 2016; Li et al., 2020), pills in 1 study (Zheng et al., 2016), and oral liquid in 1 study (Chen M. L. et al., 2021). The shortest and longest intervention durations were 2 (Xia, 2017; Chen, 2018; Wang, 2018; Wang, 2019; Fan et al., 2021) and 6 months, respectively (Kang et al., 2016; Liu, 2019; Pu et al., 2021). The characteristics of the 18 included studies are presented in Table 1 and the detailed components of CHM in Table 2.

**TABLE 1:** Characteristics of the included studies.

Study	Sample size (T/C)	Age (Y)		Gender (M/F)		Course of disease		Co-Intervention	Intervention		Duration	Outcome
		T	C	T	C	T	C		T	C		
Wang (2010)	30/30	45.8 ± 6.2	45.9 ± 7.1	25/5	22/8	8.6 ± 4.8 m	8.5 ± 4.3 m	Lifestyle intervention	CHM decoction+metformin (0.25 g,tid)	Metformin (0.25 g,tid)	3 m	①⑤⑥⑧⑩⑪
Zheng et al. (2016)	43/43	48.76 ± 5.59	49.11 ± 5.24	29/14	28/15	NR	NR	NR	CHM pill+metformin (0.5 g,tid)	Metformin (0.5 g,tid)	3 m	⑤⑥⑦⑧⑨
Kang et al. (2016)	45/45	53.59 ± 10.34	50.27 ± 10.4	28/17	26/19	2.86 ± 1.63 y	2.6 ± 1.46 y	Lifestyle intervention	CHM granules +conventional treatment	Conventional treatment (oral hypoglycemic drug and insulin)	6 m	①②③④⑤⑥⑦⑧⑨⑩⑪
Xia (2017)	30/30	51 ± 8.69	53.53 ± 7.44	18/12	17/13	7.13 ± 3.34 y	6.86 ± 3.33 y	Lifestyle intervention	CHM decoction+conventional treatment	Conventional treatment (oral hypoglycemic drug and hypolipidemic drug)	2 m	①②③④⑤⑥⑦⑧⑩⑪
Wang (2018)	30/30	51.26 ± 8.43	52.47 ± 7.52	16/14	18/12	7.15 ± 3.3 y	6.85 ± 3.41 y	Lifestyle intervention	CHM decoction+metformin and fenofibrate	Metformin and fenofibrate	2 m	①②③④⑤⑥⑦⑧⑨⑩⑪
Chen (2018)	30/30	61.07 ± 9.2	58.9 ± 10.27	12/18	12/18	3.2 ± 1.56 y	3 ± 1.39 y	Lifestyle intervention	CHM decoction+metformin (0.5 g,tid)	Metformin (0.5 g,tid)	2 m	①②③⑤⑥⑧⑨⑩⑪
Wang et al. (2018)	48/48	54.21 ± 3.71	54.27 ± 3.45	32/16	31/17	30.1 ± 4.7 m	30.6 ± 4.8 m	Lifestyle intervention	CHM decoction+saxagliptin (5 mg,qd)	Saxagliptin (5 mg,qd)	3 m	①②③④⑤⑥⑧⑪
Guan et al. (2018)	30/30	50.93 ± 9.32	53.07 ± 7.69	16/14	15/15	4.72 ± 2.91 y	5.28 ± 4.57 y	Lifestyle intervention	CHM decoction+conventional treatment	Conventional treatment (oral hypoglycemic drug and hypolipidemic drug)	3 m	①②③④⑤⑥⑧⑨⑩⑪
Zou (2019)	30/30	54 ± 6.95	51 ± 8.63	15/15	16/14	13.6 ± 5.11 y	11.1 ± 4.78 y	Lifestyle intervention	CHM decoction+metformin (0.5 g,tid) and simvastatin (10 mg,qn)	Metformin (0.5 g,tid) and simvastatin (10 mg,qn)	3 m	①②③④⑤⑥⑦⑧⑨⑩⑪
Wang (2019)	51/51	53.64 ± 5.17	54.19 ± 5.25	29/22	27/24	NR	NR	Lifestyle intervention	CHM decoction+metformin (0.5 g,tid), glimepiride (3 mg,qd), and xuezhikang capsule (0.6 g,bid)	Metformin (0.5 g,tid), glimepiride (3 mg,qd), and xuezhikang capsule (0.6 g,bid)	2 m	①⑤⑥⑧⑨⑩⑪
Liu (2019)	34/34	47.88 ± 11.94	51.41 ± 11.93	20/14	17/17	6.4 ± 5.24	7.19 ± 5.47	Lifestyle intervention	CHM decoction+ metformin (0.5 g,tid), acarbose (50 mg,tid), and rosuvastatin (10 mg,qd)	Metformin (0.5 g,tid), acarbose (50 mg,tid), and rosuvastatin (10 mg,qd)	6 m	①②③④⑤⑥⑦⑧⑨⑩⑪
Li (2019)	36/36	25–65	25–65	17/19	20/16	NR	NR	Lifestyle intervention	CHM decoction+metformin (0.85 g,qd)	Metformin (0.85 g,qd)	3 m	①②③④⑤⑥⑧⑨⑪
Wang (2020)	30/30	53.96 ± 7.21	51.96 ± 8.32	16/14	17/13	13.1 ± 4.9	12.3 ± 3.77	Lifestyle intervention	CHM decoction+metformin (0.5 g,tid) and atorvastatin (10 mg,qn)	Metformin (0.5 g,tid) and atorvastatin (10 mg,qn)	3 m	①②③④⑤⑥⑦⑧⑨⑩⑪
Liu (2020)	30/30	39.43 ± 6.31	39.1 ± 7.28	14/16	13/17	0.31 ± 0.59	0.52 ± 0.69	Lifestyle intervention	CHM decoction+metformin (0.5 g,tid), pioglitazone (30 mg,qd), and rosuvastatin (5 mg,qn)	Metformin (0.5 g,tid), pioglitazone (30 mg,qd), and rosuvastatin (5 mg,qn)	3 m	①②③④⑦⑧⑩⑪
Li et al. (2020)	40/40	55.37 ± 7.61	56.74 ± 7.95	25/15	26/14	10.42 ± 3.03	9.93 ± 2.89	Lifestyle intervention	CHM granules +metformin (1 g,bid)	Metformin (1 g,bid)	3 m	①②③④⑤⑥⑧⑨⑪
Chen et al. (2021a)	46/46	57.94 ± 9.08	58.56 ± 9.23	29/17	26/20	8.03 ± 1.76	7.45 ± 1.88	Lifestyle intervention	CHM oral liquid +conventional treatment	Conventional treatment (oral hypoglycemic drug, insulin, and atorvastatin 20 mg,qd)	3 m	①②③④⑤⑥⑧⑨⑪
Fan et al. (2021)	45/45	37.4 ± 8.1	38 ± 7.8	28/17	30/15	3.37 ± 1.4	3.54 ± 1.36	Lifestyle intervention	CHM decoction +pioglitazone and metformin tablets (15 mg:500 mg,bid) and rosuvastatin (10 mg,qn)	Pioglitazone and metformin tablets (15 mg:500 mg,bid) and rosuvastatin (10 mg,qn)	2 m	①②③⑤⑥⑦⑧⑨
Pu et al. (2021)	110/110	51.79 ± 8.77	51.78 ± 8.41	62/48	68/42	3.09 ± 0.48	3.03 ± 0.51	Lifestyle intervention	CHM decoction+metformin (0.5 g,tid), glimepiride (3 mg,qd), and xuezhikang capsule (0.6 g,bid)	Metformin (0.5 g,tid), glimepiride (3 mg,qd), and xuezhikang capsule (0.6 g,bid)	6 m	①②③④⑤⑥⑧⑨⑪

Abbreviations: T: treatment group; C: control group; CHM: Chinese herbal medicine; F: female; M: male; NR: not reported; Y: year; W: week; ①: triglyceride; ②: total cholesterol; ③: low-density lipoprotein cholesterol; ④: high-density lipoprotein cholesterol; ⑤: alanine transaminase; ⑥: aspartate transaminase; ⑦: homeostatic model assessment of insulin resistance; ⑧: fasting blood glucose; ⑨: 2-h postprandial glucose; ⑩: body mass index; ⑪: overall effective rate.

**TABLE 2:** Detailed components of CHM.

Study	Chinese herbal medicine	Ingredients of herb prescription		Usage
		Latin name	Chinese name	
Wang (2010)	Tiaozhi Huoxue Jiangtang decoction	Astragalus membranaceus (Fisch.) Bge, 15 g	Huangqi, 15 g	1 package bid
		Atractylodes chinensis (DC.) Koidz., 10 g	Cangzhu, 10 g	
		Scrophularia ningpoensis Hemsl., 15 g	Xuanshen, 15 g	
		Salvia miltiorrhiza Bunge, 20 g	Danshen, 20 g	
		Crataegus pinnatifida Bunge, 15 g	Shanzha, 15 g	
		Polygonatum kingianum Coll. et Hemsl., 15 g	Huangjing, 15 g	
		Reynoutria multiflora (Thunb.) Moldenke, 20 g	Heshouwu, 20 g	
		Euonymus alatus (Thunb.) Sieb., 15 g	Guijianyu, 15 g	
		Alisma plantago-aquatica subsp. orientale (Sam.), 15 g	Zexie, 15 g	
		Bupleurum chinense DC., 10 g	Chaihu, 10 g	
		Cyperus rotundus L., 10 g	Xiangfu, 10 g	
		Curcuma aromatica Salisb., 15 g	Yujin, 15 g	
		Paeonia lactiflora Pall. 15 g	Baishaoyao, 15 g	
		Typha angustifolia L., 10 g	Puhuang, 10 g	
		Coptis chinensis Franch., 10 g	Huanglian,10 g	
		Reynoutria japonica Houtt., 15 g	Huzhang, 15 g	
Zheng et al. (2016)	Liuwei Dihuang pill	Rehmannia glutinosa Libosch	Shudihuang	8 pills tid
		Dioscorea opposita Thunb	Shanyao	
		Cornus officinalis Sieb. et Zucc	Shanzhuyu	
		Alisma plantago-aquatica subsp. orientale (Sam.), 15 g	Zexie	
		Poria cocos (Schw.) Wolf	Fuling	
		Paeonia suffruticosa Andr	Mudanpi	
Kang et al. (2016)	Huazhuo granule	Coptis chinensis Franch	Huanglian	1 package tid
		Phellodendron amurense Rupr	Huangbo	
		Crataegus pinnatifida Bunge	Shanzha	
		Gallus gallus domesticus Brisson	Jineijin	
		Salvia miltiorrhiza Bunge	Danshen	
		Aurantii Fructus	Zhiqiao	
Xia (2017)	Compound Gegen Qinlian decoction	Pueraria montana var. thomsonii (Benth.), 15 g	Gegen, 15 g	200 ml bid
		Scutellaria baicalensis Georgi, 9 g	Huangqi, 9 g	
		Coptis chinensis Franch., 9 g	Huanglian, 9 g	
		Pseudostellaria heterophylla (Miq.) Pax, 15 g	Taizishen, 15 g	
		Poria cocos (Schw.) Wolf, 9 g	Fuling, 9 g	
		Pinellia ternata (Thunb.) Makino, 9 g	Banxia, 9 g	
		Citri Reticulatae Pericarpium, 12 g	Chenpi, 12 g	
		Bambusa tuldoides Munro, 9 g	Zhuru, 9 g	
		Carthamus tinctorius L., 12 g	Honghua, 12 g	
		Ligusticum chuanxiong Hort., 9 g	Chuanxiong, 9 g	
		Pheretima aspergillum (E.Perrier), 9 g	Dilong, 9 g	
		Atractylodes macrocephala Koidz., 12 g	Baizhu, 12 g	
Wang (2018)	Compound Gegen Qinlian decoction	Pseudostellaria heterophylla (Miq.) Pax, 15 g	Taizishen, 15 g	200 ml bid
		Atractylodes macrocephala Koidz., 12 g	Baizhu, 12 g	
		Coptis chinensis Franch., 9 g	Huanglian, 12 g	
		Pinellia ternata (Thunb.) Makino, 9 g	Banxia, 9 g	
		Poria cocos (Schw.) Wolf, 9 g	Fuling, 9 g	
		Pueraria montana var. thomsonii (Benth.), 15 g	Gegen, 15 g	
		Scutellaria baicalensis Georgi, 9 g	Huangqin, 9 g	
		Citri Reticulatae Pericarpium, 12 g	Chenpi, 12 g	
		Bambusa tuldoides Munro, 9 g	Zhuru, 9 g	
		Carthamus tinctorius L., 12 g	Honghua, 12 g	
		Pheretima aspergillum (E.Perrier), 9 g	Dilong, 9 g	
		Ligusticum chuanxiong Hort., 9 g	Chuanxiong, 9 g	
Chen (2018)	Modified Huanglian Wendan decoction	Coptis chinensis Franch., 9 g	Huanglian, 9 g	200 ml bid
		Scutellaria baicalensis Georgi, 9 g	Huangqin, 9 g	
		Trichosanthes kirilowii Maxim., 30 g	Gualou, 30 g	
		Citri Reticulatae Pericarpium, 15 g	Chenpi, 15 g	
		Pinellia ternata (Thunb.) Makino, 9 g	Banxia, 9 g	
		Bambusa tuldoides Munro, 9 g	Zhuru, 9 g	
		Fritillaria thunbergii Miq., 15 g	Zhebeimu, 15 g	
		Poria cocos (Schw.) Wolf, 15 g	Fuling, 15 g	
		Atractylodes macrocephala Koidz., 15 g	Baizhu, 15 g	
		Pueraria montana var. thomsonii (Benth.), 30 g	Gegen, 30 g	
		Salvia miltiorrhiza Bunge, 30 g	Danshen, 30 g	
		Bupleurum chinense DC., 15 g	Chaihu, 15 g	
Wang et al. (2018)	Modified Wendan decoction	Pinellia ternata (Thunb.) Makino, 20 g	Banxia, 20 g	200 ml bid
		Citrus × aurantium L., 12 g	Zhishi, 12 g	
		Citri Reticulatae Pericarpium, 15 g	Chenpi, 15 g	
		Crataegus pinnatifida Bunge, 15 g	Shanzha, 15 g	
		Bambusa tuldoides Munro, 15 g	Zhuru, 15 g	
		Salvia miltiorrhiza Bunge, 15 g	Danshen, 15 g	
		Alisma plantago-aquatica subsp. orientale (Sam.).	Zexie, 10 g	
		Poria cocos (Schw.) Wolf, 10 g	Fuling, 10 g	
		Glycyrrhiza uralensis Fisch., 6 g	Gancao, 6 g	
		Zingiber officinale Roscoe, 5 pieces	Shengjiang, 5pian	
		Ziziphus jujuba Mill., 1 piece	Dazao, 1mei	
Guan et al. (2018)	Buxin Tongmai decoction	Codonopsis pilosula (Franch.) Nannf., 20 g	Dangshen, 20 g	150 ml bid
		Angelica sinensis (Oliv.) Diels, 15 g	Danggui, 15 g	
		Ligusticum chuanxiong Hort., 10 g	Chuanxiong, 10 g	
		Achyranthes bidentata Blume, 10 g	Niuxi, 10 g	
		Rehmannia glutinosa (Gaertn.) DC., 10 g	Shengdi, 10 g	
		Astragalus membranaceus (Fisch.) Bge, 20 g	Huangqi, 20 g	
		Atractylodes macrocephala Koidz., 10 g	Baizhu, 10 g	
		Acorus calamus L., 8 g	Changpu, 8 g	
		Poria cocos (Schw.) Wolf, 8 g	Fushen, 8 g	
		Ophiopogon japonicus (Thunb.) Ker Gawl., 20 g	Maidong, 20 g	
		Trichosanthes kirilowii Maxim., 15 g	Tianhuafen, 15 g	
		Artemisia capillaris Thunb., 10 g	Yinchen, 10 g	
		Bupleurum chinense DC., 6 g	Chaihu, 6 g	
		Citrus × aurantium L., 10 g	Zhiqiao, 10 g	
Zou (2019)	Compound Gegen Qinlian decoction	Pseudostellaria heterophylla (Miq.) Pax, 15 g	Taizishen, 15 g	200 ml bid
		Pueraria montana var. thomsonii (Benth.), 15 g	Gegen, 15 g	
		Atractylodes macrocephala Koidz., 12 g	Baizhu, 12 g	
		Pinellia ternata (Thunb.) Makino, 9 g	Banxia, 9 g	
		Scutellaria baicalensis Georgi, 9 g	Huangqin, 9 g	
		Citri Reticulatae Pericarpium, 12 g	Chenpi, 12 g	
		Bambusa tuldoides Munro, 9 g	Zhuru, 9 g	
		Carthamus tinctorius L., 12 g	Honghua, 12 g	
		Pheretima aspergillum (E. Perrier), 9 g	Dilong, 9 g	
		Lycium barbarum L., 20 g	Gouqizi, 20 g	
		Ligusticum chuanxiong Hort., 9 g	Chuanxiong, 9 g	
Wang (2019)	Huoxue Jiangzhi Baogan decoction	Panax notoginseng (Burkill) F.H.Chen, 6 g	Sanqi, 6 g	150 ml bid
		Salvia miltiorrhiza Bunge, 10 g	Danshen, 10 g	
		Crataegus pinnatifida Bunge, 10 g	Shanzha, 10 g	
		Alisma plantago-aquatica subsp. orientale (Sam.), 10 g	Zexie, 10 g	
		Schisandra chinensis (Turcz.) Baill., 15 g	Wuweizi, 15 g	
		Ligusticum chuanxiong Hort., 15 g	Chuanxiong, 15 g	
		Paeonia anomala subsp. veitchii (Lynch), 15 g	Chishao, 15 g	
		Astragalus membranaceus (Fisch.) Bge, 15 g	Huangqi, 15 g	
Liu (2019)	Modified Shuilu Erxian decoction	Euryale ferox Salisb. 20 g	Qianshi, 20 g	100 ml bid
		Rosa laevigata Michx., 20 g	Jinyingzi, 20 g	
		Coptis chinensis Franch., 10 g	Huanglian, 10 g	
		Astragalus membranaceus (Fisch.) Bge, 30 g	Huangqi, 30 g	
Li (2019)	Tongtiao Tangzhi decoction	Coptis chinensis Franch., 20 g	Huanglian, 20 g	150 ml bid
		Atractylodes lancea (Thunb.) DC., 15 g	Cangzhu, 15 g	
		Codonopsis pilosula (Franch.) Nannf., 20 g	Dangshen, 20 g	
		Bupleurum chinense DC., 15 g	Chaihu, 15 g	
		Citri Reticulatae Pericarpium, 10 g	Chenpi, 10 g	
		Pinellia ternata (Thunb.) Makino, 6 g	Banxia, 6 g	
		Coix lacryma-jobi L., 20 g	Yiyiren, 20 g	
		Pueraria montana var. thomsonii (Benth.), 20 g	Gegen, 20 g	
		Litchi chinensis Sonn., 15 g	Lizhihe, 15 g	
		Crataegus pinnatifida Bunge, 20 g	Shanzha, 20 g	
		Folium Nelumbinis, 20 g	Heye, 20 g	
		Glycyrrhiza uralensis Fisch., 6 g	Gancao, 15 g	
Wang (2020)	Compound Gegen Qinlian decoction	Pseudostellaria heterophylla (Miq.) Pax, 15 g	Taizishen, 15 g	200 ml bid
		Pueraria montana var. thomsonii (Benth.), 15 g	Gegen, 15 g	
		Atractylodes macrocephala Koidz., 12 g	Baizhu, 12 g	
		Pinellia ternata (Thunb.) Makino, 9 g	Banxia, 9 g	
		Coptis chinensis Franch., 9 g	Huanglian, 9 g	
		Scutellaria baicalensis Georgi, 9 g	Huangqin, 9 g	
		Bambusa tuldoides Munro, 9 g	Zhuru, 9 g	
		Carthamus tinctorius L., 12 g	Honghua, 12 g	
		Pheretima aspergillum (E.Perrier), 9 g	Dilong, 9 g	
		Paeonia lactiflora Pall., 12 g	Shaoyao, 12 g	
		Citri Reticulatae Pericarpium, 12 g	Chenpi, 12 g	
		Ligusticum chuanxiong Hort., 9 g	Chuanxiong, 9 g	
Liu (2020)	Huazhuo Jiedu decoction	Bupleurum chinense DC.	Chaihu	100 ml bid
		Scutellaria baicalensis Georgi	Huangqin	
		Citrus × aurantium L	Zhishi	
		Paeonia lactiflora Pall	Baishao	
		Eupatorium fortunei Turcz	Peilan	
		Zingiber officinale Roscoe	Ganjiang	
		Coptis chinensis Franch	Huanglian	
		Pinellia ternata (Thunb.) Makino	Banxia	
		Bombyx mori Linnaeus	Jiangcan	
		Cryptotympana pustulata Fabr	Chantui	
		Curcuma longa L	Jianghuang	
		Rheum officinale Baill	Shudahuang	
		Glycyrrhiza uralensis Fisch	Gancao	
Li et al. (2020)	Tangzhiping granule	Morus alba L., 15 g	Sangbaipi, 15 g	1 package bid
		Alisma plantago-aquatica subsp. orientale (Sam.), 15 g	Zexie, 15 g	
		Euonymus alatus (Thunb.) Sieb., 15 g	Guijianyu, 15 g	
		Coptis chinensis Franch., 10 g	Huanglian, 10 g	
		Rheum officinale Baill., 6 g	Shudahuang, 6 g	
Chen et al. (2021b)	Dangua Humai oral liquid	Salvia miltiorrhiza Bunge	Danshen	20 ml tid
		Trichosanthes kirilowii Maxim	Gualou	
		Paeonia anomala subsp. veitchii (Lynch)	Chishao	
		Bombyx mori Linnaeus	Jiangcan	
		Pinellia ternata (Thunb.) Makino	Banxia	
		Allium chinensis G. Don	Xiebai	
Fan et al. (2021)	Gegen Qinlian decoction	Pueraria montana var. thomsonii (Benth.), 30 g	Gegen, 30 g	200 ml bid
		Scutellaria baicalensis Georgi, 15 g	Huangqin, 15 g	
		Coptis chinensis Franch., 10 g	Huanglian, 10 g	
		Processed product of Glycyrrhiza uralensis Fisch., 6 g	Zhigancao, 6 g	
Pu et al. (2021)	Jianpi Huayu Qutan decoction	Codonopsis pilosula (Franch.) Nannf., 10 g	Dangshen, 10 g	1 package tid
		Poria cocos (Schw.) Wolf, 20 g	Fuling, 20 g	
		Atractylodes macrocephala Koidz., 15 g	Baizhu, 15 g	
		Processed product of Glycyrrhiza uralensis Fisch., 6 g	Zhigancao, 6 g	
		Citri Reticulatae Pericarpium, 10 g	Chenpi, 10 g	
		Pinellia ternata (Thunb.) Makino, 10 g	Banxia, 10 g	
		Astragalus membranaceus (Fisch.) Bge, 30 g	Huangqi, 30 g	
		Rehmannia glutinosa (Gaertn.) DC., 30 g	Shengdihuang, 30 g	
		Atractylodes lancea (Thunb.) DC., 15 g	Cangzhu, 15 g	
		Scrophularia ningpoensis Hemsl., 30 g	Xuanshen, 30 g	
		Pueraria montana var. thomsonii (Benth.), 30 g	Gegen, 30 g	
		Salvia miltiorrhiza Bunge, 30 g	Danshen, 30 g	
		Crataegus pinnatifida Bunge, 15 g	Shanzha, 15 g	
		Folium Nelumbinis, 10 g	Heye, 10 g	
		Gynostemma pentaphyllum (Thunb.) Makino, 10 g	Jiaogulan, 10 g	

### 3.3 Risk of Bias Assessment

**FIGURE 2:**
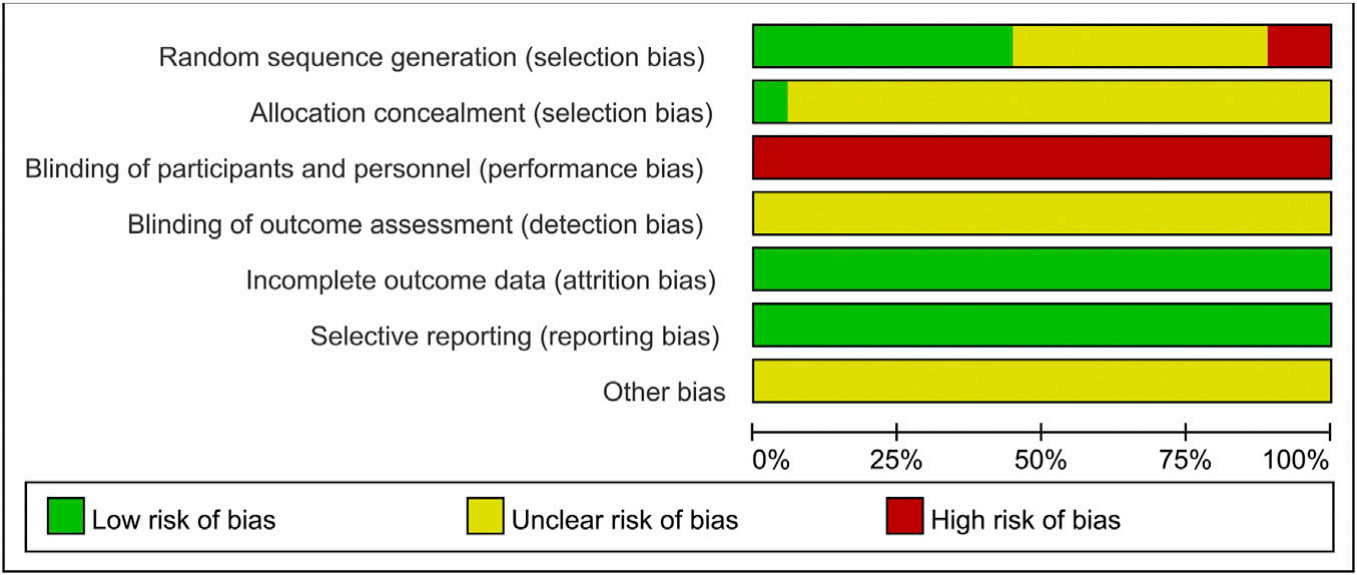
Risk of bias graph.

**FIGURE 3:**
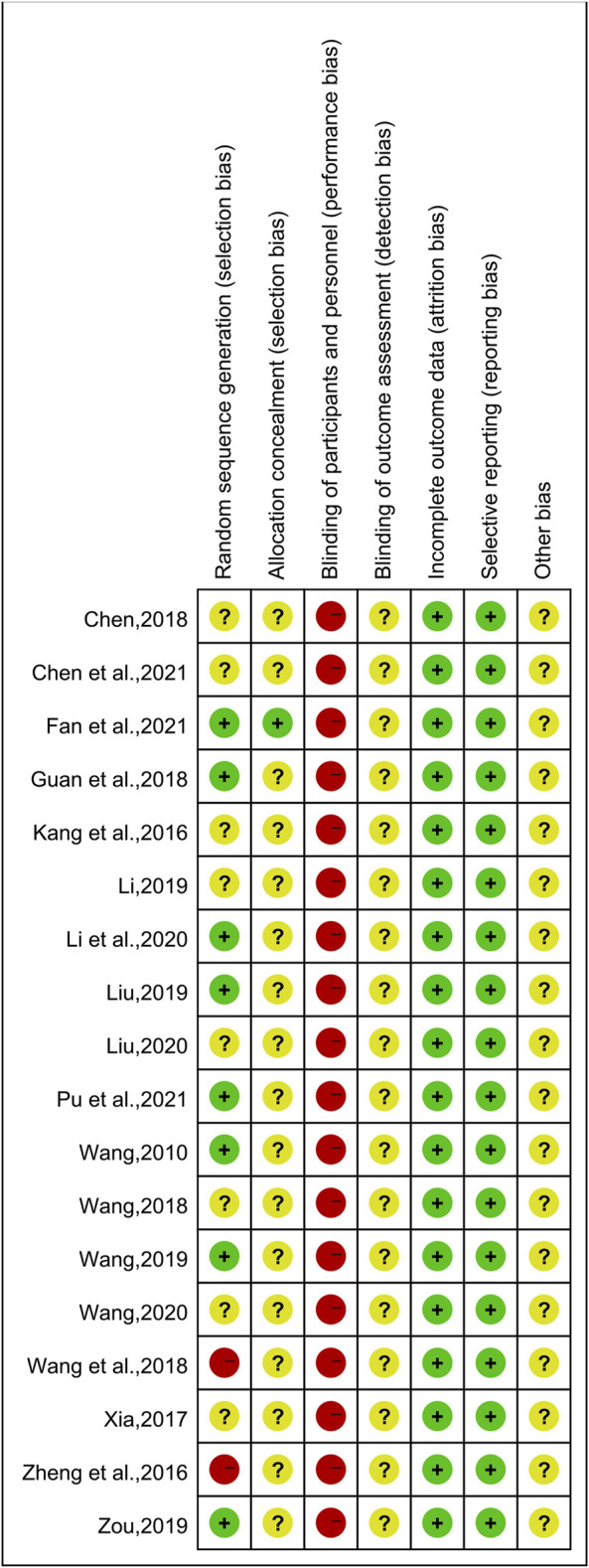
Risk of bias summary.

The results of the risk of bias assessment are shown in Figure 2 and Figure 3. Of the 18 included studies, 8 studies (Wang, 2010; Guan et al., 2018; Liu, 2019; Wang, 2019; Zou, 2019; Li et al., 2020; Fan et al., 2021; Pu et al., 2021) were classified as low risk of bias because they used the random number table for randomization. Two studies (Zheng et al., 2016; Wang et al., 2018) reported randomization according to the intervention, so they were at a high risk of bias. The other 8 studies (Kang et al., 2016; Xia, 2017; Chen, 2018; Wang, 2018; Li, 2019; Liu, 2020; Wang, 2020; Chen M. L. et al., 2021) claimed to use randomization but did not report details of randomization methodology and were therefore marked as “unclear risk.” Except for one study (Fan et al., 2021), the allocation concealment was unclear for the remaining studies. None of the experiments reported blinding of the participants or researchers. Thus, all studies were classified as having a high risk of bias in this aspect. However, these tests were considered to use objective outcome measures. With regards to other biases, none of the studies provided sufficient information that could be used in determining the presence of other significant risks of bias and thus assessed as “unclear risk.”

### 3.4 Outcomes

#### 3.4.1 Effect of Chinese Herbal Medicine on Lipid Indices

##### 3.4.1.1 Triglyceride

**FIGURE 4:**
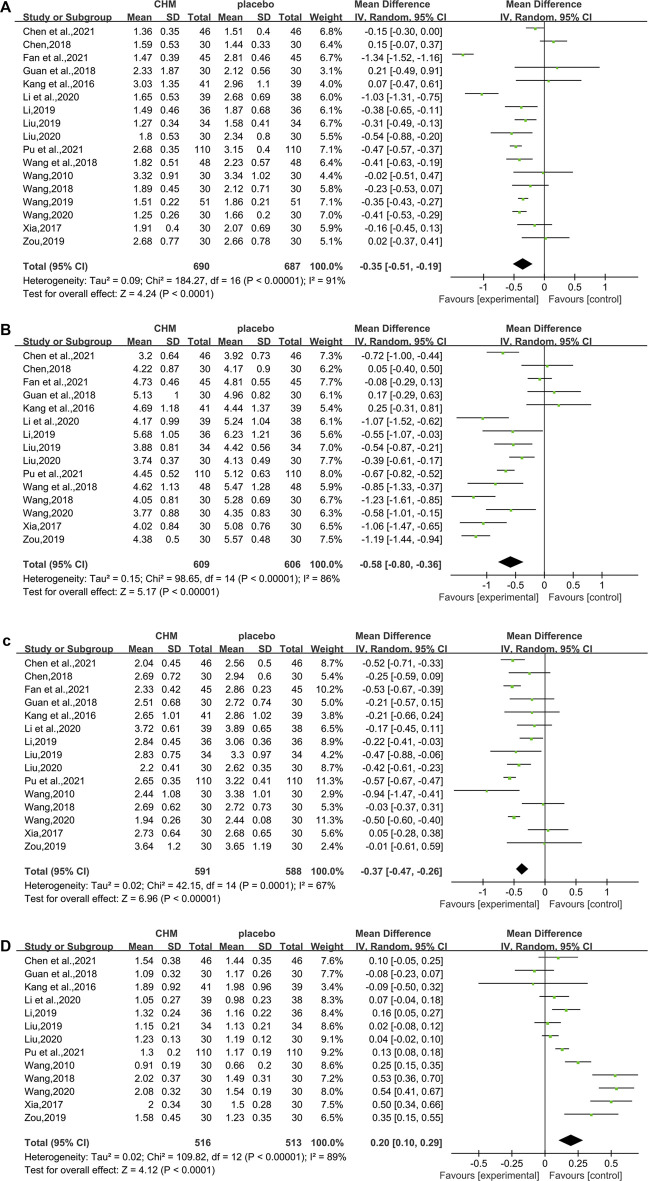
Forest plot for lipid indices. **(A) **TG; **(B)** TC; **(C)** LDL-C; and **(D)** HDL-C.

In total, 17 studies (1,377 subjects) (Wang, 2010; Kang et al., 2016; Xia, 2017; Chen, 2018; Guan et al., 2018; Wang, 2018; Wang et al., 2018; Li, 2019; Liu, 2019; Wang, 2019; Zou, 2019; Li et al., 2020; Liu, 2020; Wang, 2020; Chen M. L. et al., 2021; Fan et al., 2021; Pu et al., 2021) evaluated TG levels. Overall analyses suggested CHM combined with WM might reduce TG in T2DM with NAFLD patients (WMD = −0.35.95%CI [−0.51, −0.19],*p* < 0.0001,I^2^ = 91%, random effects model; Figure 4). With regards to subgroup analysis, there was no significant difference between different intervention durations, different types of hypoglycemic drugs, and different control treatments (*p* for interaction = 0.99, 0.95, and 0.67, respectively) (Table 3, Supplementary Figure S1).

**TABLE 3:** Subgroup analysis for outcomes.

	Number of comparisons	Result	p-value for overall effect	I^2^ (%)	p-value for subgroup difference
TG		WMD (95%CI)			
All comparisons	17	−0.35 [−0.51, −0.19]	<0.0001	91	
Intervention duration					0.99
2 m	5	−0.39 [−0.88, 0.10]	0.12	97	
3 m	9	−0.35 [−0.54, −0.16]	0.0003	80	
6 m	3	−0.35 [−0.55, −0.15]	0.0005	64	
Types of hypoglycemic drug					0.95
Metformin	11	−0.34 [−0.48, −0.21]	<0.00001	82	
Other hypoglycemic drug	6	−0.33 [−0.84, 0.18]	0.20	96	
Different control treatment					0.67
Hypoglycemic therapy	6	−0.29 [−0.66, 0.08]	0.13	89	
Hypoglycemic+hypolipidemic therapy	11	−0.38 [−0.57, −0.19]	<0.0001	93	
TC					
All comparisons	15	−0.58 [−0.80, −0.36]	<0.00001	86	
Intervention duration					0.62
2 m	4	−0.58 [−1.22, 0.07]	0.08	93	
3 m	8	−0.66 [−0.95, −0.36]	<0.0001	82	
6 m	3	−0.40 [−0.81, 0.01]	0.05	79	
Types of hypoglycemic drug					0.22
Metformin	9	−0.69 [−0.94, −0.45]	<0.00001	82	
Other hypoglycemic drug	6	−0.39 [−0.80, 0.02]	0.06	87	
Different control treatment					0.51
Hypoglycemic therapy	5	−0.44 [−0.94, 0.06]	0.08	81	
Hypoglycemic+hypolipidemic therapy	10	−0.63 [−0.89, −0.38]	<0.00001	88	
LDL-C					
All comparisons	15	−0.37 [−0.47, −0.26]	<0.00001	67	
Intervention duration					0.2
2 m	4	−0.21 [−0.52, 0.10]	0.18	81	
3 m	8	−0.38 [−0.52, −0.25]	<0.00001	61	
6 m	3	−0.51 [−0.68, −0.35]	<0.00001	21	
Types of hypoglycemic drug					0.71
Metformin	10	−0.37 [−0.50, −0.25]	<0.00001	68	
Other hypoglycemic drug	5	−0.33 [−0.54, −0.11]	0.003	70	
Different control treatment					0.05
Hypoglycemic therapy	6	−0.24 [−0.42, −0.07]	0.005	41	
Hypoglycemic+hypolipidemic therapy	9	−0.44 [−0.54, −0.34]	<0.00001	57	
HDL-C					
All comparisons	13	0.20 [0.10, 0.29]	<0.0001	89	
Intervention duration					<0.00001
2 m	2	0.51 [0.40, 0.63]	<0.00001	0	
3 m	8	0.17 [0.05, 0.30]	0.005	89	
6 m	3	0.08 [−0.02, 0.17]	0.14	56	
Types of hypoglycemic drug					0.55
Metformin	9	0.22 [0.12, 0.32]	<0.0001	90	
Other hypoglycemic drug	4	0.12 [−0.17, 0.41]	0.40	90	
Different control treatment					0.35
Hypoglycemic therapy	4	0.15 [0.05, 0.25]	0.004	58	
Hypoglycemic+hypolipidemic therapy	9	0.23 [0.10, 0.35]	0.0005	92	
ALT					
All comparisons	17	−4.99 [−6.64, −3.33]	<0.00001	83	
Intervention duration					0.37
2 m	5	−2.60 [−6.67, 1.47]	0.21	84	
3 m	9	−6.06 [−8.72, −3.41]	<0.00001	83	
6 m	3	−5.33 [−8.56, −2.09]	0.001	89	
Types of hypoglycemic drug					0.71
Metformin	11	−4.87 [−6.58, −3.16]	<0.00001	77	
Other hypoglycemic drug	6	−5.74 [−9.98, −1.51]	0.008	90	
Different control treatment					0.002
Hypoglycemic therapy	7	−7.55 [−9.33, −5.77]	<0.00001	64	
Hypoglycemic+hypolipidemic therapy	10	−2.91 [−5.28, −0.53]	0.02	84	
AST					
All comparisons	17	−4.76 [−6.35, −3.16]	<0.00001	86	
Intervention duration					0.62
2 m	5	−4.33 [−6.57, −2.09]	0.0001	62	
3 m	9	−5.59 [−8.32, −2.87]	<0.0001	90	
6 m	3	−3.51 [−6.97, −0.04]	0.05	91	
Types of hypoglycemic drug					0.46
Metformin	11	−4.42 [−6.19, −2.66]	<0.00001	84	
Other hypoglycemic drug	6	−5.86 [−9.23, −2.48]	0.0007	87	
Different control treatment					0.15
Hypoglycemic therapy	7	−6.10 [−8.58, −3.62]	<0.00001	89	
Hypoglycemic+hypolipidemic therapy	10	−3.73 [−5.76, −1.70]	0.0003	81	
HOMA-IR					
All comparisons	9	−1.01 [−1.22, −0.79]	<0.00001	72	
Intervention duration					0.25
2 m	3	−0.83 [−1.23, −0.44]	<0.0001	68	
3 m	4	−0.94 [−1.21, −0.68]	<0.00001	63	
6 m	2	−1.39 [−1.94, −0.84]	<0.00001	68	
Types of hypoglycemic drug					0.82
Metformin	6	−0.98 [−1.18, −0.78]	<0.00001	52	
Other hypoglycemic drug	3	−1.06 [−1.75, −0.38]	0.002	89	
Different control treatment					0.45
Hypoglycemic therapy	2	−1.26 [−2.04, −0.48]	0.002	88	
Hypoglycemic+hypolipidemic therapy	7	−0.95 [−1.19, −0.70]	<0.00001	67	
FPG					
All comparisons	18	−0.87 [−1.13, −0.61]	<0.00001	88	
Intervention duration					0.67
2 m	5	−1.02 [−1.37, −0.67]	<0.00001	78	
3 m	10	−0.83 [−1.25, −0.41]	0.0001	91	
6 m	3	−0.74 [−1.39, −0.10]	0.02	87	
Types of hypoglycemic drug					0.50
Metformin	12	−0.95 [−1.23, −0.67]	<0.00001	88	
Other hypoglycemic drug	6	−0.70 [−1.36, −0.05]	0.03	90	
Different control treatment					0.80
Hypoglycemic therapy	7	−0.91 [−1.24, −0.57]	<0.00001	63	
Hypoglycemic+hypolipidemic therapy	11	−0.84 [−1.19, −0.49]	<0.00001	92	
2hPG					
All comparisons	14	−1.45 [−2.00, −0.91]	<0.00001	92	
Intervention duration					0.78
2 m	4	−1.55 [−2.87, −0.24]	0.02	95	
3 m	7	−1.30 [−2.11, −0.49]	0.002	92	
6 m	3	−1.70 [−2.48, −0.92]	<0.0001	68	
Types of hypoglycemic drug					0.73
Metformin	10	−1.51 [−2.17, −0.86]	<0.00001	93	
Other hypoglycemic drug	4	−1.29 [−2.40, −0.17]	0.02	88	
Different control treatment					0.47
Hypoglycemic therapy	5	−1.74 [−2.75, −0.73]	0.0007	94	
Hypoglycemic+hypolipidemic therapy	9	−1.29 [−1.98, −0.61]	0.0002	91	
BMI					
All comparisons	11	−0.73 [−1.35, −0.12]	0.02	73	
Intervention duration					0.001
2 m	4	0.04 [−0.63, 0.72]	0.90	63	
3 m	5	−0.96 [−1.80, −0.12]	0.02	51	
6 m	2	−2.11 [−3.03, −1.19]	<0.00001	0	
Types of hypoglycemic drug					0.81
Metformin	8	−0.65 [−1.29, −0.02]	0.04	64	
Other hypoglycemic drug	3	−0.90 [−2.78, 0.98]	0.35	88	
Different control treatment					0.01
Hypoglycemic therapy	3	−2.07 [−3.46, −0.68]	0.003	72	
Hypoglycemic+hypolipidemic therapy	8	−0.22 [−0.71, 0.27]	0.39	44	

##### 3.4.1.2 Total Cholesterol

In total, 15 studies (1,215 patients) (Kang et al., 2016; Xia, 2017; Chen, 2018; Guan et al., 2018; Wang, 2018; Wang et al., 2018; Li, 2019; Liu, 2019; Zou, 2019; Li et al., 2020; Liu, 2020; Wang, 2020; Chen M. L. et al., 2021; Fan et al., 2021; Pu et al., 2021) mentioned TC as a biomarker. A significant difference was found between the two groups (WMD = −0.58. 95%CI [−0.80, −0.36], *p* < 0.00001, I^2^ = 86%, random effects model; Figure 4). Subgroup analyses were carried out according to different intervention durations and types of hypoglycemic drugs, and control treatments showed no significant difference in intervention effect between groups (*p* for interaction = 0.62, 0.22, and 0.51, respectively), and significant heterogeneity was seen (Table 3, Supplementary Figure S1).

##### 3.4.1.3 Low-Density Lipoprotein Cholesterol

In total, 15 studies (Wang, 2010; Kang et al., 2016; Xia, 2017; Chen, 2018; Guan et al., 2018; Wang, 2018; Li, 2019; Liu, 2019; Zou, 2019; Li et al., 2020; Liu, 2020; Wang, 2020; Chen M. L. et al., 2021; Fan et al., 2021; Pu et al., 2021) involving 1,179 T2DM patients with NAFLD demonstrated a therapeutic effect of adding CHM to conventional therapy on LDL-C. The results showed a significant lowering effect of CHM plus WM treatment on LDL-C levels (WMD = −0.37, 95%CI [−0.47, −0.26], *p* < 0.00001, I^2^ = 67%, random effects model; Figure 4). Subgroup analysis showed no significant difference between subgroups of different intervention durations (*p* = 0.20), different types of hypoglycemic drugs (*p* = 0.71), and different control treatments (*p* = 0.05) (Table 3, Supplementary Figure S1).

##### 3.4.1.4 High Density Liptein Cholesterol

The HDL-C was assessed in 13 studies (1,029 patients) (Wang, 2010; Kang et al., 2016; Xia, 2017; Guan et al., 2018; Wang, 2018; Li, 2019; Liu, 2019; Zou, 2019; Li et al., 2020; Liu, 2020; Wang, 2020; Chen M. L. et al., 2021; Pu et al., 2021). The significant differences between groups were found, which revealed that the combination of CHM and WM provided superior benefit (WMD = 0.20, 95%CI [0.10, 0.29], *p* < 0.0001, I^2^ = 89%, random effects model; Figure 4). Subgroup analysis showed that there was a significant difference in different intervention durations (*p* < 0.00001), and no significant difference between different types of hypoglycemic drugs (*p* = 0.55) and control treatment (*p* = 0.35) was observed (Table 3, Supplementary Figure S1).

#### 3.4.2 Effect of Chinese Herbal Medicine on Liver Functions

##### 3.4.2.1 Alanine Transaminase

**FIGURE 5:**
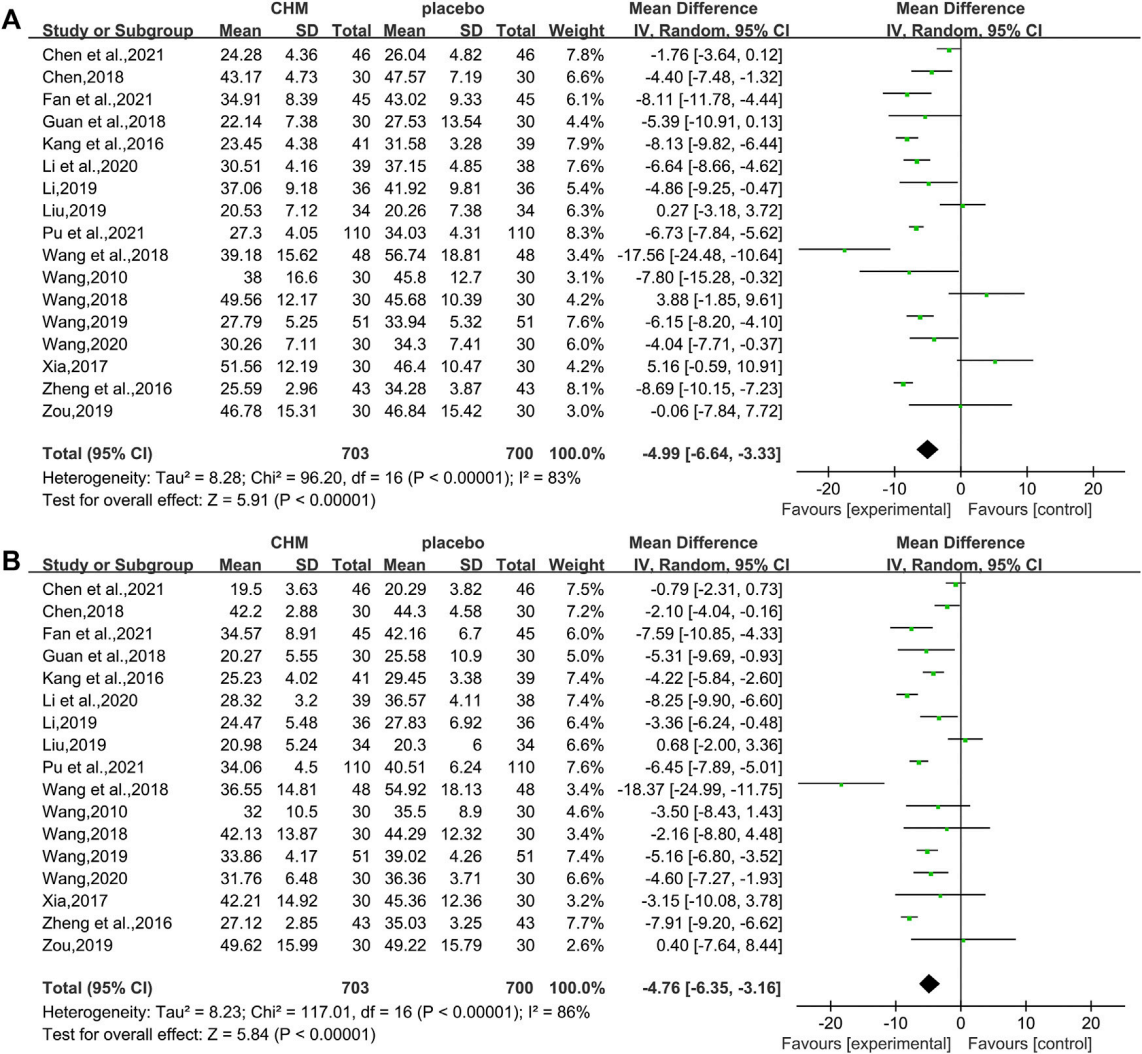
Forest plot for liver functions. **(A)** ALT and **(B)** AST.

In total, 17 studies (1,403 patients) (Wang, 2010; Kang et al., 2016; Zheng et al., 2016; Xia, 2017; Chen, 2018; Guan et al., 2018; Wang, 2018; Wang et al., 2018; Li, 2019; Liu , 2019; Wang, 2019; Zou, 2019; Li et al., 2020; Wang, 2020; Chen M. L. et al., 2021; Fan et al., 2021; Pu et al., 2021) mentioned ALT as a biomarker. Results from the meta-analyses indicated that CHM plus WM showed more benefits in reducing ALT levels than WM alone (WMD = −4.99, 95%CI [−6.64, −3.33], *p* < 0.00001, I^2^ = 83%, random effects model; Figure 5). Results of subgroup analyses indicated a significant difference in different control treatments (*p* = 0.002), while no significant difference was observed between subgroups of different intervention durations (*p* = 0.37) and different types of hypoglycemic drugs (*p* = 0.71) (Table 3, Supplementary Figure S1).

##### 3.4.2.2 Aspartate Transaminase

Of the 18 studies, 17 studies (1,403 patients) (Wang, 2010; Kang et al., 2016; Zheng et al., 2016; Xia, 2017; Chen, 2018; Guan et al., 2018; Wang, 2018; Wang et al., 2018; Li, 2019; liu, 2019; Wang, 2019; Zou, 2019; Li et al., 2020; Wang, 2020; Chen M. L. et al., 2021; Fan et al., 2021; Pu et al., 2021) reported the effectiveness of CHM on AST when compared with WM alone. Noteworthy lowering on AST was observed after treatments (WMD = −4.76, 95%CI [−6.35, −3.16], *p* < 0.00001, I^2^ = 86%, random effects model; Figure 5). Subgroup analyses by different intervention durations, types of hypoglycemic drugs, and control treatments showed no significant difference in effect size (*p* for interaction = 0.62, 0.46, and 0.15 respectively). (Table 3, Supplementary Figure S1).

#### 3.4.3 Effect of Chinese Herbal Medicine on Insulin and Glycemic Indices

##### 3.4.3.1 Homeostatic Model Assessment of Insulin Resistance

**FIGURE 6:**
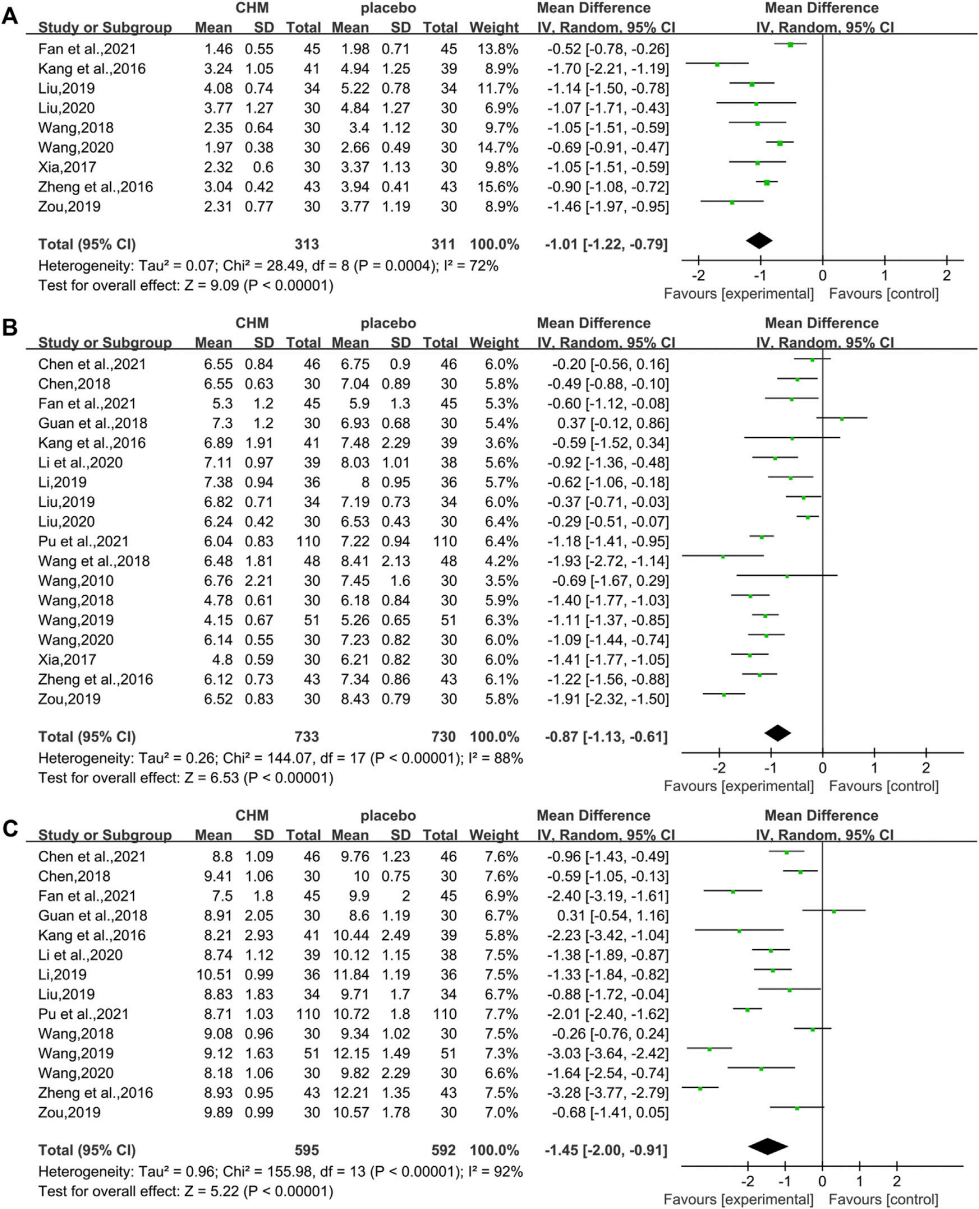
Forest plot for insulin and glycemic indices. **(A)** HOMA-IR; **(B)** FBG; and **(C)** 2hPG.

The HOMA-IR was assessed in 9 studies (624 patients) (Kang et al., 2016; Zheng et al., 2016; Xia, 2017; Wang, 2018; Liu , 2019; Zou, 2019; Liu, 2020; Wang, 2020; Fan et al., 2021). The analysis exhibited a significant difference between two groups (WMD = −1.01, 95%CI [−1.22, −0.79], *p* < 0.00001, I^2^ = 72%, random effects model; Figure 6). Obviously, CHM combined with WM was significantly superior to WM alone in improving HOMA-IR. With regards to subgroup analysis, no significant difference was found in different intervention durations (*p* = 0.25), different types of hypoglycemic drugs (*p* = 0.82), and different control treatments (*p* = 0.45) (Table 3, Supplementary Figure S1).

##### 3.4.3.2 Fasting Blood Glucose

All the 18 studies (1,463 patients) (Wang, 2010; Kang et al., 2016; Zheng et al., 2016; Xia, 2017; Chen, 2018; Guan et al., 2018; Wang, 2018; Wang et al., 2018; Li, 2019; Liu, 2019; Wang, 2019; Zou, 2019; Li et al., 2020; Liu, 2020; Wang, 2020; Chen M. L. et al., 2021; Fan et al., 2021; Pu et al., 2021) mentioned the changes in FBG. Overall analyses revealed that a combination of CHM plus WM showed more effective in reducing FBG levels than WM alone (WMD = −0.87, 95%CI [−1.13, −0.61], *p* < 0.00001, I^2^ = 88%, random effects model; Figure 6). As to subgroup analyses, we observed no significant difference between subgroups of different intervention durations, types of hypoglycemic drugs, and control treatments (*p* for interaction = 0.67, 0.50 and 0.80 respectively). (Table 3, Supplementary Figure S1).

##### 3.4.3.3 Two-Hour Postprandial Glucose

In total, 14 studies (Kang et al., 2016; Zheng et al., 2016; Chen, 2018; Guan et al., 2018; Wang, 2018; Li, 2019; Liu , 2019; Wang, 2019; Zou, 2019; Li et al., 2020; Wang, 2020; Chen M. L. et al., 2021; Fan et al., 2021; Pu et al., 2021) reported 2hPG level, with a total of 1.187 patients. A noteworthy reduction of 2hPG was observed by CHM plus WM compared with WM (WMD = −1.45.95%CI [−2.00, −0.91], *p* < 0.00001, I^2^ = 92%, random effects model; Figure 6). Results of subgroup analyses revealed that there was no significant difference in different intervention durations (*p* = 0.78), different types of hypoglycemic drugs (*p* = 0.73), and different control treatments (*p* = 0.47) (Table 3, Supplementary Figure S1).

#### 3.4.4 Effect of CHM on the Body Mass Index

**FIGURE 7:**
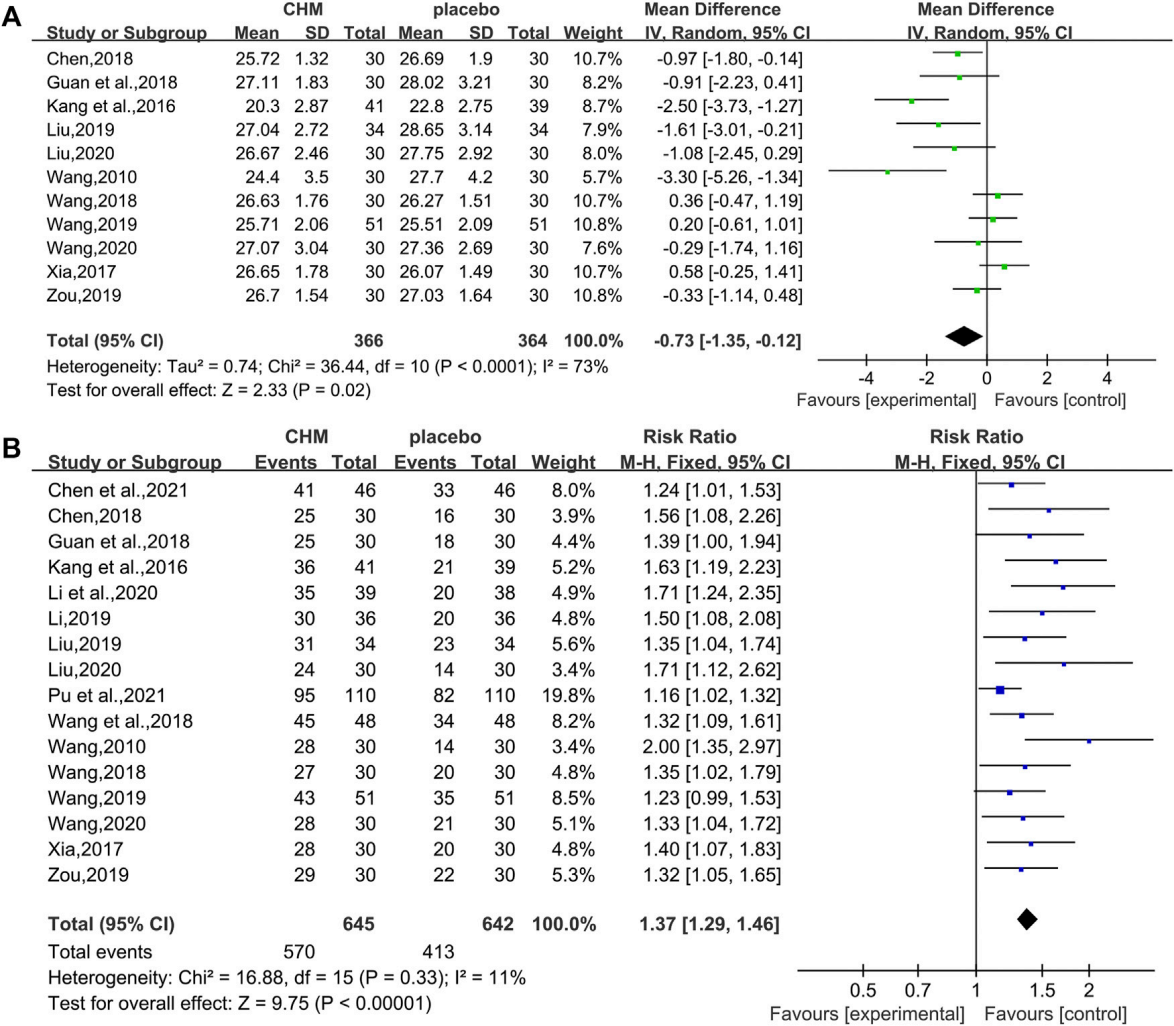
Forest plot for the body mass index and overall effective rate. **(A)** BMI and **(B)** overall effective rate.

In total, 11 RCTs (730 patients) (Wang, 2010; Kang et al., 2016; Xia, 2017; Chen, 2018; Guan et al., 2018; Wang, 2018; Liu , 2019; Wang, 2019; Zou, 2019; Liu, 2020; Wang, 2020) were evaluated for the effectiveness of CHM on BMI. Compared with WM, CHM combined with WM could reduce the BMI level (WMD = −0.73.95%CI [−1.35, −0.12], *p* = 0.02, I^2^ = 73%, random effects model; Figure 7). Subgroup analyses showed there was a significant difference in different intervention durations (*p* = 0.001) and different control treatments (*p* = 0.01), while no significant difference was detected in different types of hypoglycemic drugs (*p* = 0.81). (Table 3, Supplementary Figure S1).

#### 3.4.5 Overall Effective Rate and Adverse Effects of CHM

##### 3.4.5.1 Overall Effective Rate

In total, 16 studies (1,287 patients) (Wang, 2010; Kang et al., 2016; Xia, 2017; Chen, 2018; Guan et al., 2018; Wang, 2018; Wang et al., 2018; Li, 2019; Liu , 2019; Wang, 2019; Zou, 2019; Li et al., 2020; , 2020; Wang, 2020; Chen M. L. et al., 2021; Pu et al., 2021) reported overall effective rate. The combined effects suggested a significant improving effect of CHM plus WM on the overall effective rate in T2DM with NAFLD patients when compared with WM alone (RR = 1.37.95%CI [1.29, 1.46], *p* < 0.00001, I^2^ = 11%, fixed effects model; Figure 7).

##### 3.4.5.2 Adverse Effects

A total of 13 RCTs reported adverse effects. Among them, 6 literatures (Chen, 2018; Guan et al., 2018; Li, 2019; liu, 2019; Liu , 2020; Chen M. L. et al., 2021) indicated that no adverse events occurred during the treatment period, while 7 literatures (Kang et al., 2016; Xia, 2017; Wang, 2018; Wang et al., 2018; Zou, 2019; Li et al., 2020; Wang, 2020) reported that general adverse events occurred. The most common adverse effect was abdominal fullness (Kang et al., 2016; Xia, 2017; Wang, 2018; Zou, 2019; Li et al., 2020; Wang, 2020) followed by diarrhea (Xia, 2017; Wang, 2018; Zou, 2019; Li et al., 2020; Wang, 2020). Adverse effects such as nausea, anorexia, and dizziness have also been reported in these studies. However, all adverse effects resolved (or disappeared) after symptomatic treatment.

### 3.4.6 Publication Bias

**FIGURE 8:**
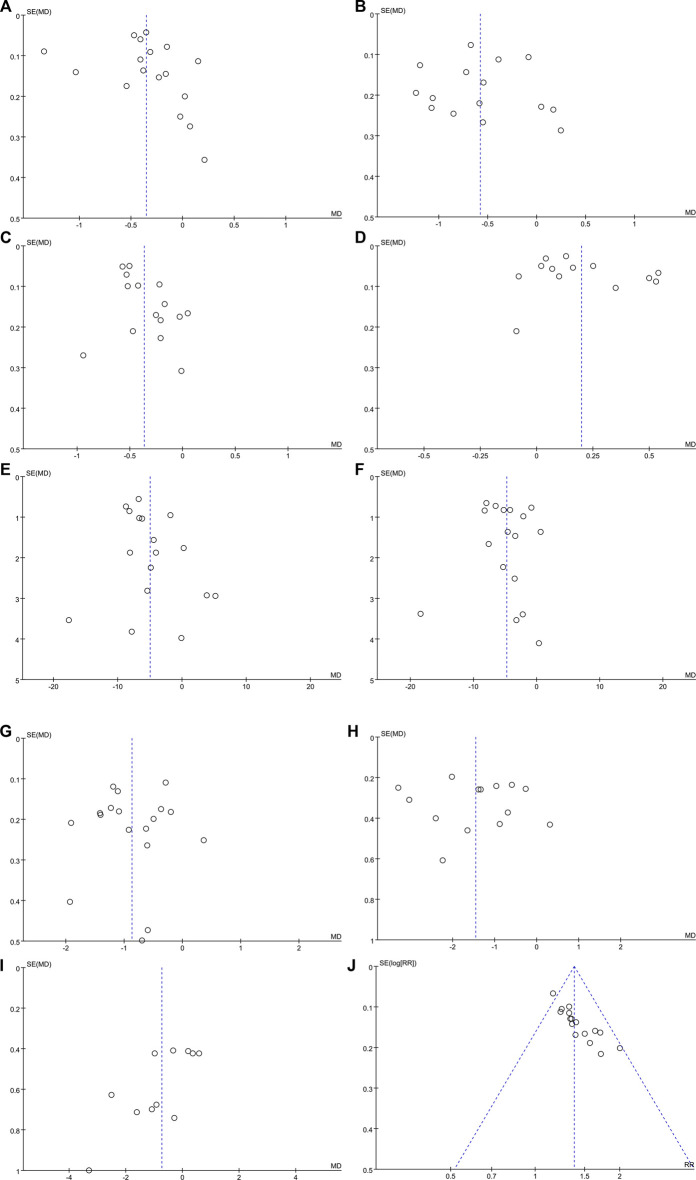
Funnel plots for assessing publication bias. **(A)** TG; **(B)** TC; **(C)** LDL-C; **(D)** HDL-C; **(E)** ALT; **(F)** AST; **(G)** FBG; **(H)** 2hPG; **(I)** BMI; and **(J)** overall effective rate.

Funnel plots and Egger’s test were performed to evaluate publication bias. The funnel plot (Figures 8C,I,J) revealed a slight asymmetry and Egger’s test indicated possible publication bias in LDL-C (t = 2.75, *p* = 0.016),BMI(t = −3.04, *p* = 0.014), and overall effective rate (t = 8.02, *p* < 0.001) (Supplementary Figure S2). Figures 8A, B, D–H show inverted and symmetrical funnels. The results of Egger’s test were as follows: TG (t = 0.54, *p* = 0.599), TC (t = 0.12, *p* = 0.909), HDL-C (t = 1.32, *p* = 0.213), ALT (t = 1.56, *p* = 0.139), AST (t = 0.44, *p* = 0.666), FBG (t = −0.22, *p* = 0.830), and 2hPG (t = 0.23, *p* = 0.821) (Supplementary Figure S2), indicating that these outcomes had no significant publication bias.

### 3.4.7 Sensitivity Analysis

After excluding each study, we found no significant changes in the results, and all the results showed good agreement. For further validation, we used STATA v14.0 and performed sensitivity analysis of TG, ALT, FBG, and overall effective rate; the results were considered robust (Supplementary Figure S3).

## 4 Discussion

### 4.1 Summary of the Main Results

For this study, a total of 783 relevant articles were retrieved, and 18 articles were included in the meta-analysis after screening. The main findings of our meta-analysis showed that when compared with WM therapy alone, a combination of CHM and WM therapy was effective for improvement of lipid and glucose metabolism, liver function, insulin resistance, and body mass. The high level of heterogeneity could be attributable to different interventions. The subgroup analysis was performed based on different intervention durations and different types of WM to explain or reduce the degree of associated heterogeneity and obtain a more reliable conclusion. Subgroup analyses showed that intervention duration was primarily responsible for the positive changes in HDL-C and BMI. A few studies did not report adverse effects. The methodological quality of the included studies was poor by the risk assessment of bias. Sensitivity analysis indicated that the results were robust.

### 4.2 Quality of Evidence

In this study, we used GRADEpro to assess the quality of evidence. The assessment showed that the evidence of all the results was of low quality, except that the evidence quality of the overall effective rate was moderate (Table 4). The decreased certainty of the evidence was mainly attributed to the low methodological quality and the high level of heterogeneity among the studies. Therefore, the results of this study should be applied cautiously to clinical practice, and more high-quality RCTs are needed to evaluate efficacy.

**TABLE 4:** Certainty of evidence: CHM compared to control treatment for T2DM with NAFLD.

Certainty assessment							No. of patients		Effect		Certainty	Importance
No. of Studies	Study design	Risk of bias	Inconsistency	Indirectness	Imprecision	Other considerations	CHM	Control treatment	Relative (95% CI)	Absolute (95% CI)		
Triglyceride												
17	Randomized trials	Serious a	Serious b	Not serious	Not serious	None	690	687	-	MD 0.35 lower (0.51 lower to 0.19 lower)	⊕⊕⊚⊚Low	Important
Total cholesterol												
15	Randomized trials	Serious a	Serious b	Not serious	Not serious	None	609	606	-	MD 0.58 SD lower (0.8 lower to 0.36 lower)	⊕⊕⊚⊚Low	Important
Low-density lipoprotein cholesterol												
15	Randomized trials	Serious a	Serious b	Not serious	Not serious	None	591	588	-	MD 0.37 lower (0.47 lower to 0.26 lower)	⊕⊕⊚⊚Low	Important
High-density lipoprotein cholesterol												
13	Randomized trials	Serious a	Serious b	Not serious	Not serious	None	516	513	-	MD 0.2 higher (0.1 higher to 0.29 higher)	⊕⊕⊚⊚Low	Important
Alanine transaminase												
17	Randomized trials	Serious a	Serious b	Not serious	Not serious	None	703	700	-	MD 4.99 lower (6.64 lower to 3.33 lower)	⊕⊕⊚⊚Low	Important
Aspartate transaminase												
17	Randomized trials	Serious a	Serious b	Not serious	Not serious	None	703	700	-	MD 4.76 lower (6.35 lower to 3.16 lower)	⊕⊕⊚⊚Low	Important
Homeostatic model assessment of insulin resistance												
9	Randomized trials	Serious a	Serious b	Not serious	Not serious	None	313	311	-	MD 1.01 lower (1.22 lower to 0.79 lower)	⊕⊕⊚⊚Low	Important
Fasting blood glucose												
18	Randomized trials	Serious a	Serious b	Not serious	Not serious	None	733	730	-	MD 0.87 lower (1.13 lower to 0.61 lower)	⊕⊕⊚⊚Low	Important
Two-hour postprandial glucose												
14	Randomized trials	Serious a	Serious b	Not serious	Not serious	None	595	592	-	MD 1.45 lower (2 lower to 0.91 lower)	⊕⊕⊚⊚Low	Important
Body mass index												
11	Randomized trials	Serious a	Serious b	Not serious	Not serious	None	366	364	-	MD 0.73 lower (1.35 lower to 0.12 lower)	⊕⊕⊚⊚Low	Important
Overall effective rate												
16	Randomized trials	Serious a	Not serious	Not serious	Not serious	None	570/645 (88.4%)	413/642 (64.3%)	RR 1.37 (1.29–1.46)	238 more per 1,000 (from 187 more to 296 more)	⊕⊕⊕⊚Moderate	Important

Abbreviations: CI, confidence interval; MD, mean difference; RR, risk ratio; a, the risk of bias assessment is mostly “unclear risk” in articles; b, ihere is serious heterogeneity among the studies included in the analysis of this outcome.

### 4.3 Frequency Distribution Analysis of Chinese Herb Medicines

A total of 70 single CHMs were recorded, sorted by frequency of occurrence from high to low, thereafter listed the CHMs with a frequency of more than 5 times (Table 5). The top four were *Coptis chinensis Franch (huáng lián)*, *Pinellia ternata (Thunb.) Makino (bàn xià)*, *Pueraria montana* var. *thomsonii (Benth.) (gě gēn)*, and *Citri Reticulatae Pericarpium (chén pí)*.

**TABLE 5:** Frequency of CHM (more than 5 times).

No.	Chinese herb	Latin name	Frequency
1	Huang Lian (huáng lián)	Coptis chinensis Franch	11
2	Ban Xia (bàn xià)	Pinellia ternata (Thunb.) Makino	10
3	Ge Gen (gě gēn)	Pueraria montana var. thomsonii (Benth.)	8
4	Chen Pi (chén pí)	Citri Reticulatae Pericarpium	8
5	Bai Zhu (bái zhú)	Atractylodes macrocephala Koidz	7
6	Dan Shen (dān shēn)	Salvia miltiorrhiza Bunge	7
7	Fu Ling (fú líng)	Poria cocos (Schw.) Wolf	7
8	Huang Qin (huáng qín)	Scutellaria baicalensis Georgi	7
9	Shan Zha (shān zhā)	Crataegus pinnatifida Bunge	6
10	Chuan Xiong (chuān xiōng)	Ligusticum chuanxiong Hort	6
11	Zhu Ru (zhú rú)	Bambusa tuldoides Munro	6
12	Huang Qi (huáng qí)	Astragalus membranaceus (Fisch.) Bge	5
13	Chai Hu (chái hú)	Bupleurum chinense DC.	5
14	Ze Xie (zé xiè)	Alisma plantago-aquatica subsp. orientale (Sam.)	5

For *Coptis chinensis Franch.*, which has the highest frequency of occurrence, pharmacological studies have shown that berberine is the main active ingredient, which has definite effects of reducing hepatocyte lipid accumulation, anti-inflammation, and anti-fibrosis (Li et al., 2021), lowering blood glucose and improving insulin resistance (Chen Y. et al., 2021), and providing typical multi-target and multi-system pharmacological effects on T2DM with NAFLD. Berberine can regulate hepatic metabolism by protecting the intestinal mucosal epithelial barrier, thereby regulating the microenvironment of the intestinal microbiota and changing microbiota-derived metabolites such as short-chain fatty acids and secondary bile acids (Betrapally et al., 2017; Sun et al., 2017; Tian et al., 2019). Berberine can also promote GLP-1 secretion to improve glucose metabolism by up-regulating the related expression of proglucagon and prohormone convertase mRNA (Yu et al., 2010). With regards to *Pueraria montana* var. *thomsonii (Benth.)*, its main active ingredients are isoflavones, including puerarin and daidzin. Studies have shown that puerarin can repair liver injury and reduce dyslipidemia caused by liver fat deposition by inhibiting IκBα/NF-κB p65 signaling axis activity (Hu et al., 2021). Furthermore, it can improve insulin resistance and oxidative stress by affecting insulin receptor signaling pathways and adjusting the structure of the intestinal microbiome (Zhang H. M. et al., 2019).

### 4.4 Strengths and Limitations for Research

This study was conducted in strict accordance with the method of systematic review, and we interpreted the results cautiously to avoid confusion while ensuring accuracy. We found that combined CHM therapy might improve lipid and glucose metabolism, liver function and insulin resistance, and reduce body weight as well as increase overall effective rate, better than conventional therapy. Therefore, the results may provide new treatment opportunities, new ideas, and new directions for the study of T2DM with NAFLD.

However, this review has a few limitations that warrant discussion. For example, due to the incomplete nature of information provided by most articles as well as the flawed study design, the overall methodological quality of the included studies is poor, which may lead to overestimation of efficacy. Therefore results should be interpreted cautiously.

Second, while we performed the subgroup analysis, the source of heterogeneity could not be determined completely. Heterogeneity could have emerged due to the different composition and dose of CHM used in interventions and different dosage forms of CHM (such as decoction, tablets, granules, and pills). In addition, since some studies did not report adverse effects in the aftermath of CHM treatment, the safety associated with CHM remains unclear, and further studies are needed to confirm it. Finally, T2DM with NAFLD as a metabolic disease, lifestyle intervention (such as physical exercise and diet) and pharmacological treatment are the important therapy modalities and have a great impact on values of the biomarkers evaluated. In this study, lifestyle intervention and pharmacological treatment were only briefly statistically analyzed in Table 1, so subsequent studies can further explore the effects of different physical exercises, diets, and pharmacological treatments on the basis of this study, for example, physical exercise, to analyze whether physical exercise is combined, and the specific method and duration of physical exercise. This allows a more detailed and in-depth discussion of T2DM with NAFLD. In the meantime, it is necessary and important to compare whether the effect of two herbs differ on T2DM with NAFLD and T2DM without NAFLD. This is also a significant direction in our subsequent study.

## 5 Conclusion

In summary, the CHM in combination with WM seems to be more beneficial in T2DM with NAFLD patients in improving lipid and glucose metabolism, liver function, and insulin resistance as well as improving overall efficiency and reducing body weight. Given the poor quality of reports from these studies and uncertain evidence, these findings should be interpreted cautiously. Future RCTs with larger samples and higher quality should be conducted to provide more accurate and complete data to support and validate the clinical efficacy and safety of CHM in the treatment of T2DM with NAFLD.

## Data Availability

The original contributions presented in the study are included in the article/Supplementary Material; further inquiries can be directed to the corresponding author.

## Abbreviations

2hPG, two-hour postprandial glucose; ALT, alanine transaminase; AST, aspartate transaminase; BMI, body mass index; CHM: Chinese herbal medicine; CIs, confidence intervals; CNKI, China National Knowledge Infrastructure; FBG, fasting blood glucose; GRADE, grading of recommendations assessment, development, and evaluation; HDL-C, high-density lipoprotein cholesterol; IR, insulin resistance; HOMA-IR, homeostatic model assessment of insulin resistance; LDL-C: low-density lipoprotein cholesterol; NAFLD, nonalcoholic fatty liver disease; PRISMA-P, Preferred Reporting Items for Systematic Review and Meta-Analysis Protocols; RCTs, randomized controlled trials; T2DM, type 2 diabetes mellitus; TC, total cholesterol; TG: triglyceride; RR, risk ratio; TCM, traditional Chinese medicine; WM: Western medicine; WMDs, weighted mean differences.
