# Supplementary material for: Chinese Herbal Medicine for Type 2 Diabetes Mellitus With Nonalcoholic Fatty Liver Disease: A Systematic Review and Meta-Analysis
Source: Front Pharmacol. 2022 Jun 27;13:863839. doi: 10.3389/fphar.2022.863839 (PMC9271569; doi:10.3389/fphar.2022.863839)
Supplement: Supplementary file 1 [file DataSheet1.docx]

Supplementary Material

[Supplementary Table S1 Search strategy 2](#_Toc94122688)

[Supplementary File S1 The PRISMA checklist of this meta-analysis 7](#_Toc94122689)

[Supplementary Figure S1 Subgroup analysis 10](#_Toc94122690)

[Supplementary Figure S2 Egger’s plot 15](#_Toc94122691)

[Supplementary Figure S3 Sensitivity analysis 17](#_Toc94122692)

# Supplementary Table S1 Search strategy

| **Databases** | **Search items** | **Number** |
| --- | --- | --- |
| Pubmed | ((((((((((((((((((Non-alcoholic Fatty Liver Disease[MeSH Terms]) OR (Non alcoholic Fatty Liver Disease[Title/Abstract])) OR (NAFLD[Title/Abstract])) OR (Nonalcoholic Fatty Liver Disease[Title/Abstract])) OR (Fatty Liver, Nonalcoholic[Title/Abstract])) OR (Fatty Livers, Nonalcoholic[Title/Abstract])) OR (Liver, Nonalcoholic Fatty[Title/Abstract])) OR (Livers, Nonalcoholic Fatty[Title/Abstract])) OR (Nonalcoholic Fatty Liver[Title/Abstract])) OR (Nonalcoholic Fatty Livers[Title/Abstract])) OR (Nonalcoholic Steatohepatitis[Title/Abstract])) OR (Nonalcoholic Steatohepatitides[Title/Abstract])) OR (Steatohepatitides, Nonalcoholic[Title/Abstract])) OR (Steatohepatitis, Nonalcoholic[Title/Abstract]))) AND ((((((((((((((((((((((((((((((((Diabetes Mellitus, Type 2[MeSH Terms]) OR (Diabetes Mellitus, Noninsulin-Dependent[Title/Abstract])) OR (Diabetes Mellitus, Ketosis-Resistant[Title/Abstract])) OR (Diabetes Mellitus, Ketosis Resistant[Title/Abstract])) OR (Ketosis-Resistant Diabetes Mellitus[Title/Abstract])) OR (Diabetes Mellitus, Non Insulin Dependent[Title/Abstract])) OR (Diabetes Mellitus, Non-Insulin-Dependent[Title/Abstract])) OR (Non-Insulin-Dependent Diabetes Mellitus[Title/Abstract])) OR (Diabetes Mellitus, Stable[Title/Abstract])) OR (Stable Diabetes Mellitus[Title/Abstract])) OR (Diabetes Mellitus, Type II[Title/Abstract])) OR (NIDDM[Title/Abstract])) OR (Diabetes Mellitus, Noninsulin Dependent[Title/Abstract])) OR (Diabetes Mellitus, Maturity-Onset[Title/Abstract])) OR (Diabetes Mellitus, Maturity Onset[Title/Abstract])) OR (Maturity-Onset Diabetes Mellitus[Title/Abstract])) OR (Maturity Onset Diabetes Mellitus[Title/Abstract])) OR (MODY[Title/Abstract])) OR (Diabetes Mellitus, Slow-Onset[Title/Abstract])) OR (Diabetes Mellitus, Slow Onset[Title/Abstract])) OR (Slow-Onset Diabetes Mellitus[Title/Abstract])) OR (Type 2 Diabetes Mellitus[Title/Abstract])) OR (Noninsulin-Dependent Diabetes Mellitus[Title/Abstract])) OR (Noninsulin Dependent Diabetes Mellitus[Title/Abstract])) OR (Maturity-Onset Diabetes[Title/Abstract])) OR (Diabetes, Maturity-Onset[Title/Abstract])) OR (Maturity Onset Diabetes[Title/Abstract])) OR (Type 2 Diabetes[Title/Abstract])) OR (Diabetes, Type 2[Title/Abstract])) OR (Diabetes Mellitus, Adult-Onset[Title/Abstract])) OR (Adult-Onset Diabetes Mellitus[Title/Abstract])) OR (Diabetes Mellitus, Adult Onset[Title/Abstract]))) AND (((((((((((((((Medicine, Chinese Traditional[MeSH Terms]) OR (Traditional Chinese Medicine[Title/Abstract])) OR (Chung I Hsueh[Title/Abstract])) OR (Hsueh, Chung I[Title/Abstract])) OR (Traditional Medicine, Chinese[Title/Abstract])) OR (Zhong Yi Xue[Title/Abstract])) OR (Chinese Traditional Medicine[Title/Abstract])) OR (Chinese Medicine, Traditional[Title/Abstract])) OR (Traditional Tongue Diagnosis[Title/Abstract])) OR (Tongue Diagnoses, Traditional[Title/Abstract])) OR (Tongue Diagnosis, Traditional[Title/Abstract])) OR (Traditional Tongue Diagnoses[Title/Abstract])) OR (Traditional Tongue Assessment[Title/Abstract])) OR (Tongue Assessment, Traditional[Title/Abstract])) OR (Traditional Tongue Assessments[Title/Abstract])) | 13 |
| Cochrane  Library | #1 MeSH descriptor: [Non-alcoholic Fatty Liver Disease] explode all trees  #2 (Non alcoholic Fatty Liver Disease):ti,ab,kw OR (NAFLD):ti,ab,kw OR (Nonalcoholic Fatty Liver Disease):ti,ab,kw OR (Fatty Liver, Nonalcoholic):ti,ab,kw OR (Liver, Nonalcoholic Fatty):ti,ab,kw (Word variations have been searched)  #3 (Nonalcoholic Fatty Liver):ti,ab,kw OR (Nonalcoholic Fatty Livers):ti,ab,kw OR (Nonalcoholic Steatohepatitis):ti,ab,kw OR (Steatohepatitis, Nonalcoholic):ti,ab,kw OR (Steatohepatitides, Nonalcoholic):ti,ab,kw (Word variations have been searched)  #4 #1 OR #2 OR #3  #5 MeSH descriptor: [Diabetes Mellitus, Type 2] explode all trees  #6 (Diabetes Mellitus, Noninsulin-Dependent):ti,ab,kw OR (Diabetes Mellitus, Ketosis-Resistant):ti,ab,kw OR (Diabetes Mellitus, Ketosis Resistant):ti,ab,kw OR (Diabetes Mellitus, Non Insulin Dependent):ti,ab,kw OR (Diabetes Mellitus, Non-Insulin-Dependent):ti,ab,kw (Word variations have been searched)  #7 (Non-Insulin-Dependent Diabetes Mellitus):ti,ab,kw OR (Diabetes Mellitus, Stable):ti,ab,kw OR (Stable Diabetes Mellitus):ti,ab,kw OR (Diabetes Mellitus, Type II):ti,ab,kw OR (NIDDM):ti,ab,kw (Word variations have been searched)  #8 (Diabetes Mellitus, Noninsulin Dependent):ti,ab,kw OR (Diabetes Mellitus, Maturity-Onset):ti,ab,kw OR (Diabetes Mellitus, Maturity Onset):ti,ab,kw OR (Diabetes Mellitus, Slow-Onset):ti,ab,kw OR (Type 2 Diabetes Mellitus):ti,ab,kw (Word variations have been searched)  #9 (Noninsulin-Dependent Diabetes Mellitus):ti,ab,kw OR (Noninsulin Dependent Diabetes Mellitus):ti,ab,kw OR (Maturity-Onset Diabetes):ti,ab,kw OR (Type 2 Diabetes):ti,ab,kw OR (Diabetes Mellitus, Adult-Onset):ti,ab,kw (Word variations have been searched)  #10 #5OR#6OR#7OR#8OR#9  #11 MeSH descriptor: [Medicine, Chinese Traditional] explode all trees  #12 (Traditional Chinese Medicine):ti,ab,kw OR (Chung I Hsueh):ti,ab,kw OR (Traditional Medicine, Chinese):ti,ab,kw OR (Zhong Yi Xue):ti,ab,kw OR (Chinese Traditional Medicine):ti,ab,kw (Word variations have been searched)  #13 (Traditional Tongue Diagnosis):ti,ab,kw OR (Tongue Diagnosis, Traditional):ti,ab,kw OR (Traditional Tongue Assessment):ti,ab,kw OR (Traditional Tongue Assessments):ti,ab,kw (Word variations have been searched)  #14 #11OR#12OR#13  #15 #4AND#10AND#14 | 1 |
| EMBASE | #1 'nonalcoholic fatty liver'/exp  #2 'nafld':ab,ti OR 'nonalcoholic fatty liver disease':ab,ti OR 'fatty liver, nonalcoholic':ab,ti OR 'non alcoholic fatty liver disease':ab,ti OR 'non-alcoholic fatty liver disease':ab,ti OR 'nonalcoholic steatohepatitis':ab,ti OR 'steatohepatitis, nonalcoholic':ab,ti  #3 'non insulin dependent diabetes mellitus'/exp  #4 'diabetes mellitus, type 2':ab,ti OR 'diabetes mellitus, noninsulin-dependent':ab,ti OR 'diabetes mellitus, ketosis-resistant':ab,ti OR 'diabetes mellitus, ketosis resistant':ab,ti OR 'ketosis-resistant diabetes mellitus':ab,ti OR 'diabetes mellitus, non insulin dependent':ab,ti OR 'non-insulin-dependent diabetes mellitus':ab,ti OR 'diabetes mellitus, stable':ab,ti OR 'stable diabetes mellitus':ab,ti OR 'diabetes mellitus, type ii':ab,ti OR 'diabetes mellitus, noninsulin dependent':ab,ti OR 'diabetes mellitus, maturity-onset':ab,ti OR 'mody':ab,ti OR 'diabetes mellitus, slow-onset':ab,ti OR 'diabetes mellitus, slow onset':ab,ti OR 'type 2 diabetes mellitus':ab,ti OR 'noninsulin dependent diabetes mellitus':ab,ti OR 'maturity-onset diabetes':ab,ti OR 'maturity onset diabetes':ab,ti OR 'type 2 diabetes':ab,ti OR 'diabetes, type 2':ab,ti OR 'diabetes mellitus, adult-onset':ab,ti  #5 #1 OR #2  #6 #3 OR #4  #7 #5 AND #6  #8 'chinese medicine'/exp  #9 'medicine, chinese traditional':ab,ti OR 'traditional chinese medicine':ab,ti OR 'chung i hsueh':ab,ti OR 'hsueh, chung i':ab,ti OR 'traditional medicine, chinese':ab,ti OR 'zhong yi xue':ab,ti OR 'chinese traditional medicine':ab,ti OR 'chinese medicine, traditional':ab,ti OR 'traditional tongue diagnosis':ab,ti OR 'tongue diagnoses, traditional':ab,ti OR 'traditional tongue assessment':ab,ti OR 'herbal medicine':ab,ti  #10 #8 OR #9  #11 #7 AND #10 | 40 |
| Web of Science | #1 TS=(Non-alcoholic Fatty Liver Disease) OR AB= (NAFLD OR Nonalcoholic Fatty Liver Disease OR Fatty Liver*, Nonalcoholic OR Liver*, Nonalcoholic Fatty OR Nonalcoholic Fatty Liver* OR Nonalcoholic Steatohepatiti* OR Steatohepatiti*, Nonalcoholic)  #2 TS= (Diabetes Mellitus, Type 2) OR AB= (Diabetes Mellitus, Noninsulin-Dependent OR Diabetes Mellitus, Ketosis*Resistant OR Diabetes Mellitus, Non*Insulin Dependent OR Diabetes Mellitus, Stable OR Diabetes Mellitus, Type II OR NIDDM OR Diabetes Mellitus, Maturity*Onset OR MODY OR Diabetes Mellitus, Slow*Onset OR Type 2 Diabetes Mellitus OR Noninsulin*Dependent Diabetes Mellitus OR Diabetes Mellitus, Adult Onset)  #3 TS= (Medicine, Chinese Traditional) OR AB= (Traditional Chinese Medicine OR Chung I Hsueh OR Zhong Yi Xue OR Chinese Traditional Medicine OR Chinese Medicine, Traditional OR Traditional Tongue Diagnosis OR Tongue Diagnos*, Traditional OR Traditional Tongue Assessment*)  #4 #3 AND #2 AND #1 | 7 |
| CNKI | (SU='非酒精性脂肪性肝病'+'代谢相关脂肪性肝病') AND (SU='2型糖尿病'+'糖尿病'+'T2DM'+'消渴') AND (FT='中医'+'中药'+'中医药'+'中西医'+'中草药''+‘中成药’) | 315 |
| Wanfang  Data | (主题:("非酒精性脂肪性肝病" or "代谢相关脂肪性肝病") or 题名或关键词:("非酒精性脂肪性肝病" or "代谢相关脂肪性肝病") or 摘要:("非酒精性脂肪性肝病" or "代谢相关脂肪性肝病")) and (主题:("2型糖尿病" or "糖尿病" or"T2DM" or "消渴" ) or 题名或关键词:("2型糖尿病" or "糖尿病" or"T2DM" or "消渴" ) or 摘要:("2型糖尿病" or "糖尿病" or"T2DM" or "消渴" )) and (主题:("中医" or "中药" or "中医药" or "中西医" or "中草药" or "中成药" )or 题名或关键词:("中医" or "中药" or "中医药" or "中西医" or "中草药" or "中成药" ) or 摘要:("中医" or "中药" or "中医药" or "中西医" or "中草药" or "中成药" )) | 139 |
| VIP | ((M=非酒精性脂肪性肝病 OR 代谢相关脂肪性肝病) OR (K=非酒精性脂肪性肝病 OR 代谢相关脂肪性肝病) OR (R=非酒精性脂肪性肝病 OR 代谢相关脂肪性肝病)) AND ((M=2型糖尿病 OR 糖尿病 OR T2DM OR 消渴) OR (K=2型糖尿病 OR 糖尿病 OR T2DM OR 消渴) OR (R=2型糖尿病 OR 糖尿病 OR T2DM OR 消渴)) AND ((M=中医 OR 中药 OR 中医药 OR 中西医 OR 中草药 OR 中成药) OR (K=中医 OR 中药 OR 中医药 OR 中西医 OR 中草药 OR 中成药) OR (R=中医 OR 中药 OR 中医药 OR 中西医 OR 中草药 OR 中成药)) | 79 |
| CBM | [("非酒精性脂肪性肝病"[摘要:智能] OR "代谢相关脂肪性肝病"[摘要:智能]) AND ("2型糖尿病"[摘要:智能] OR "糖尿病"[摘要:智能] OR "消渴"[摘要:智能] OR "T2DM"[摘要:智能]) AND ("中医"[全部字段:智能] OR "中药"[全部字段:智能] OR "中医药"[全部字段:智能] OR "中西医"[全部字段:智能] OR "中草药"[全部字段:智能] OR "中成药"[全部字段:智能])](javascript:toDoRelimitSearch();) | 188 |

# Supplementary File S1 The PRISMA checklist of this meta-analysis

| **Section/topic** | **#** | **Checklist item** | **Reported on page #** |
| --- | --- | --- | --- |
| **TITLE** | | |  |
| Title | 1 | Identify the report as a systematic review, meta-analysis, or both. | 1 |
| **ABSTRACT** | | |  |
| Structured summary | 2 | Provide a structured summary including, as applicable: background; objectives; data sources; study eligibility criteria, participants, and interventions; study appraisal and synthesis methods; results; limitations; conclusions and implications of key findings; systematic review registration number. | 1-2 |
| **INTRODUCTION** | | |  |
| Rationale | 3 | Describe the rationale for the review in the context of what is already known. | 2-4 |
| Objectives | 4 | Provide an explicit statement of questions being addressed with reference to participants, interventions, comparisons, outcomes, and study design (PICOS). | 4 |
| **METHODS** | | |  |
| Protocol and registration | 5 | Indicate if a review protocol exists, if and where it can be accessed (e.g., Web address), and, if available, provide registration information including registration number. | 4,8;  CRD42021271488 |
| Eligibility criteria | 6 | Specify study characteristics (e.g., PICOS, length of follow-up) and report characteristics (e.g., years considered, language, publication status) used as criteria for eligibility, giving rationale. | 8 |
| Information sources | 7 | Describe all information sources (e.g., databases with dates of coverage, contact with study authors to identify additional studies) in the search and date last searched. | 8 |
| Search | 8 | Present full electronic search strategy for at least one database, including any limits used, such that it could be repeated. | 8; Supplement Table S1 |
| Study selection | 9 | State the process for selecting studies (i.e., screening, eligibility, included in systematic review, and, if applicable, included in the meta-analysis). | 8 |
| Data collection process | 10 | Describe method of data extraction from reports (e.g., piloted forms, independently, in duplicate) and any processes for obtaining and confirming data from investigators. | 8-9 |
| Data items | 11 | List and define all variables for which data were sought (e.g., PICOS, funding sources) and any assumptions and simplifications made. | 8 |
| Risk of bias in individual studies | 12 | Describe methods used for assessing risk of bias of individual studies (including specification of whether this was done at the study or outcome level), and how this information is to be used in any data synthesis. | 8 |
| Summary measures | 13 | State the principal summary measures (e.g., risk ratio, difference in means). | 8-9 |
| Synthesis of results | 14 | Describe the methods of handling data and combining results of studies, if done, including measures of consistency (e.g., I^2^) for each meta-analysis. | 8-9 |

| **Section/topic** | **#** | **Checklist item** | **Reported on page #** |
| --- | --- | --- | --- |
| Risk of bias across studies | 15 | Specify any assessment of risk of bias that may affect the cumulative evidence (e.g., publication bias, selective reporting within studies). | 8-9 |
| Additional analyses | 16 | Describe methods of additional analyses (e.g., sensitivity or subgroup analyses, meta-regression), if done, indicating which were pre-specified. | 9 |
| **RESULTS** | | |  |
| Study selection | 17 | Give numbers of studies screened, assessed for eligibility, and included in the review, with reasons for exclusions at each stage, ideally with a flow diagram. | 9; Figure1 |
| Study characteristics | 18 | For each study, present characteristics for which data were extracted (e.g., study size, PICOS, follow-up period) and provide the citations. | 9-10; Table1 |
| Risk of bias within studies | 19 | Present data on risk of bias of each study and, if available, any outcome level assessment (see item 12). | 10; Figure2 |
| Results of individual studies | 20 | For all outcomes considered (benefits or harms), present, for each study: (a) simple summary data for each intervention group (b) effect estimates and confidence intervals, ideally with a forest plot. | 10; Figure3 |
| Synthesis of results | 21 | Present results of each meta-analysis done, including confidence intervals and measures of consistency. | 10-18; Figure4-7 |
| Risk of bias across studies | 22 | Present results of any assessment of risk of bias across studies (see Item 15). | 18; Figure8 |
| Additional analysis | 23 | Give results of additional analyses, if done (e.g., sensitivity or subgroup analyses, meta-regression [see Item 16]). | 18 |
| **DISCUSSION** | | |  |
| Summary of evidence | 24 | Summarize the main findings including the strength of evidence for each main outcome; consider their relevance to key groups (e.g., healthcare providers, users, and policy makers). | 18-20 |
| Limitations | 25 | Discuss limitations at study and outcome level (e.g., risk of bias), and at review-level (e.g., incomplete retrieval of identified research, reporting bias). | 20-21 |
| Conclusions | 26 | Provide a general interpretation of the results in the context of other evidence, and implications for future research. | 21 |
| **FUNDING** | | |  |
| Funding | 27 | Describe sources of funding for the systematic review and other support (e.g., supply of data); role of funders for the systematic review. | 21 |

*From:*  Moher D, Liberati A, Tetzlaff J, Altman DG, The PRISMA Group (2009). Preferred Reporting Items for Systematic Reviews and Meta-Analyses: The PRISMA Statement. PLoS Med 6(6): e1000097. doi:10.1371/journal.pmed1000097

For more information, visit: **www.prisma-statement.org**

# Supplementary Figure S1 Subgroup analysis

1.Subgroup analysis for TG and TC.


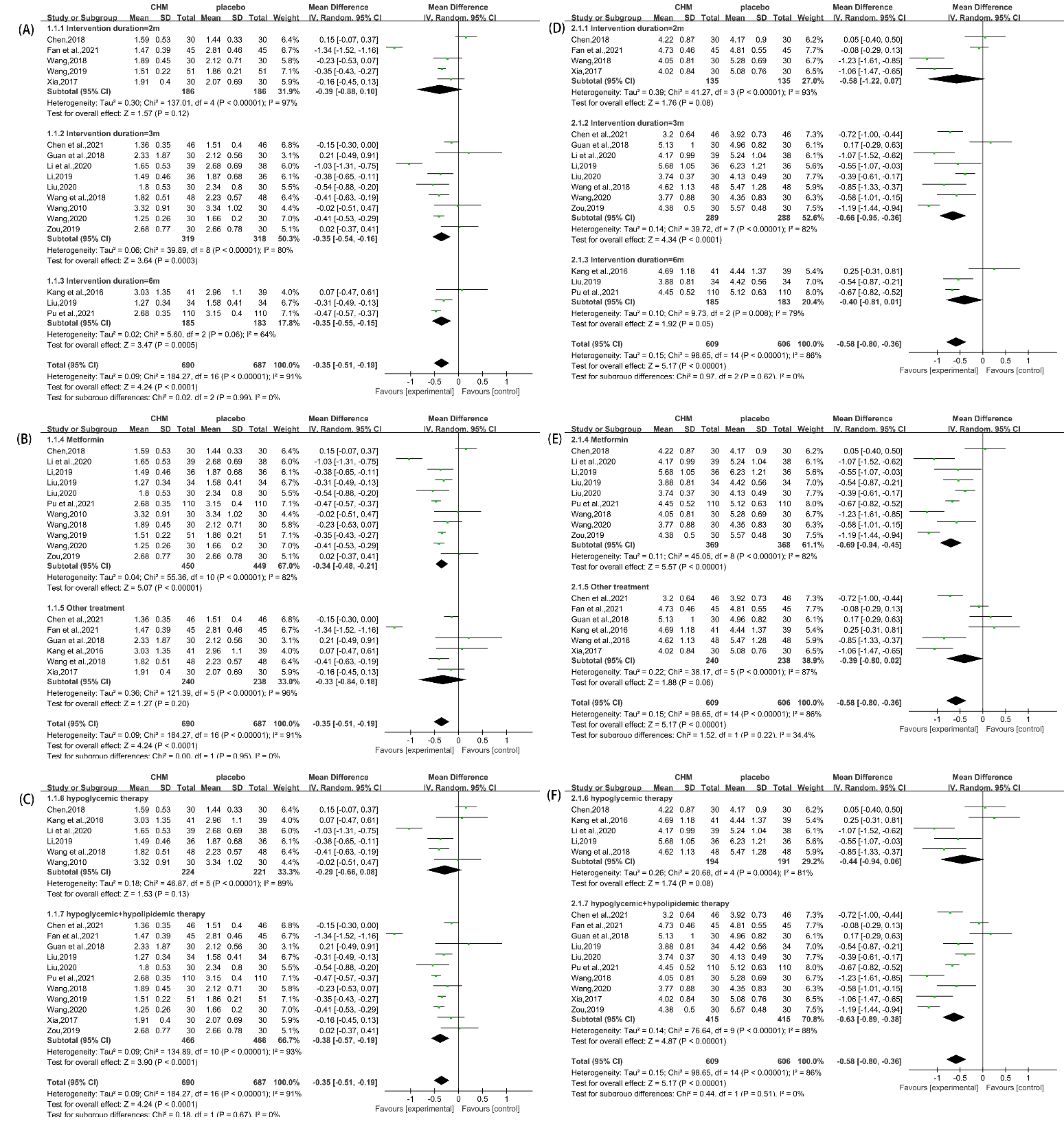
(A)-(C): subgroup analysis for TG. (D)-(F): subgroup analysis for TC.

2.Subgroup analysis for LDL-C and HDL-C.


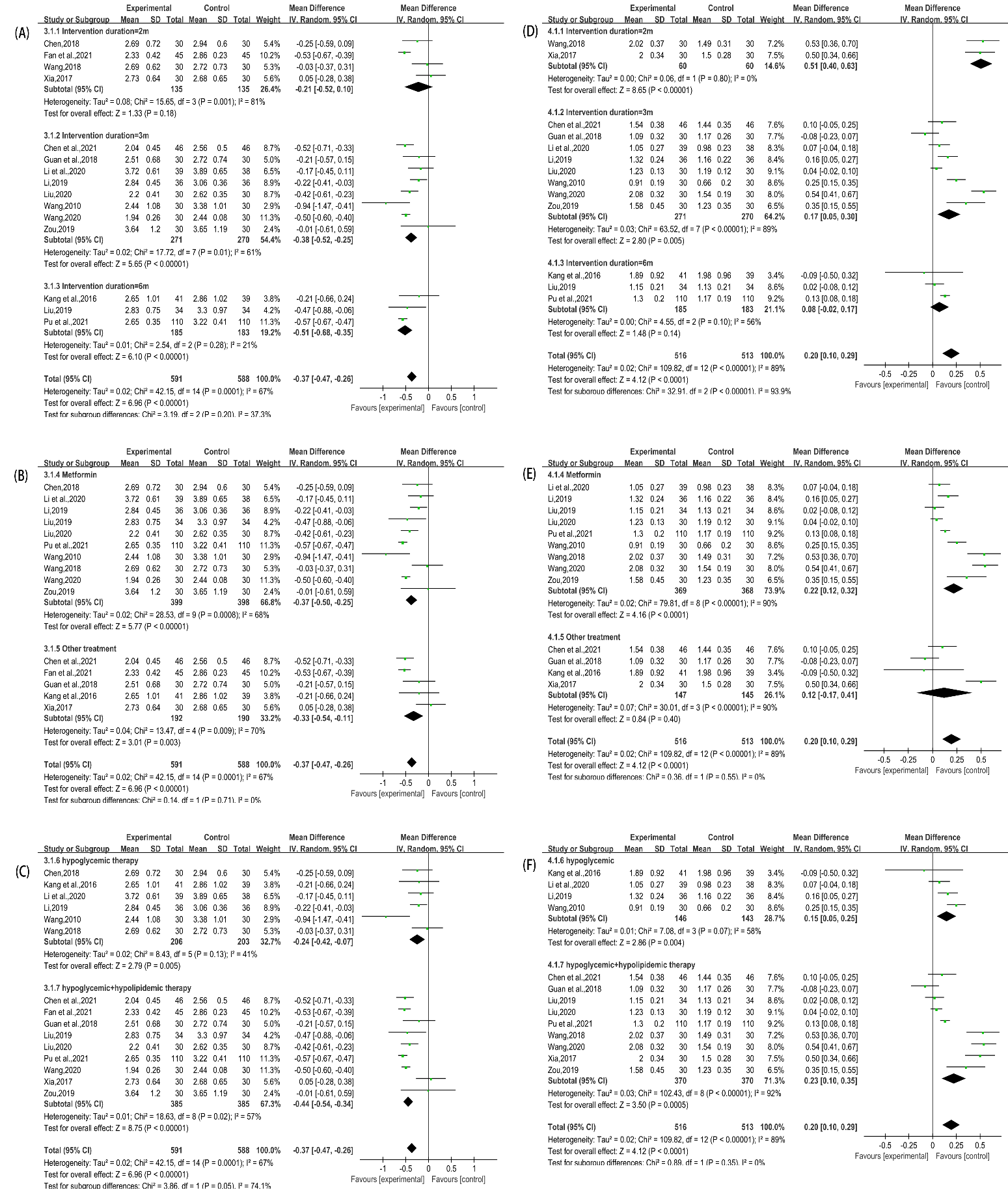
(A)-(C): subgroup analysis for LDL-C. (D)-(F): subgroup analysis for HDL-C.

3.Subgroup analysis for ALT and AST.


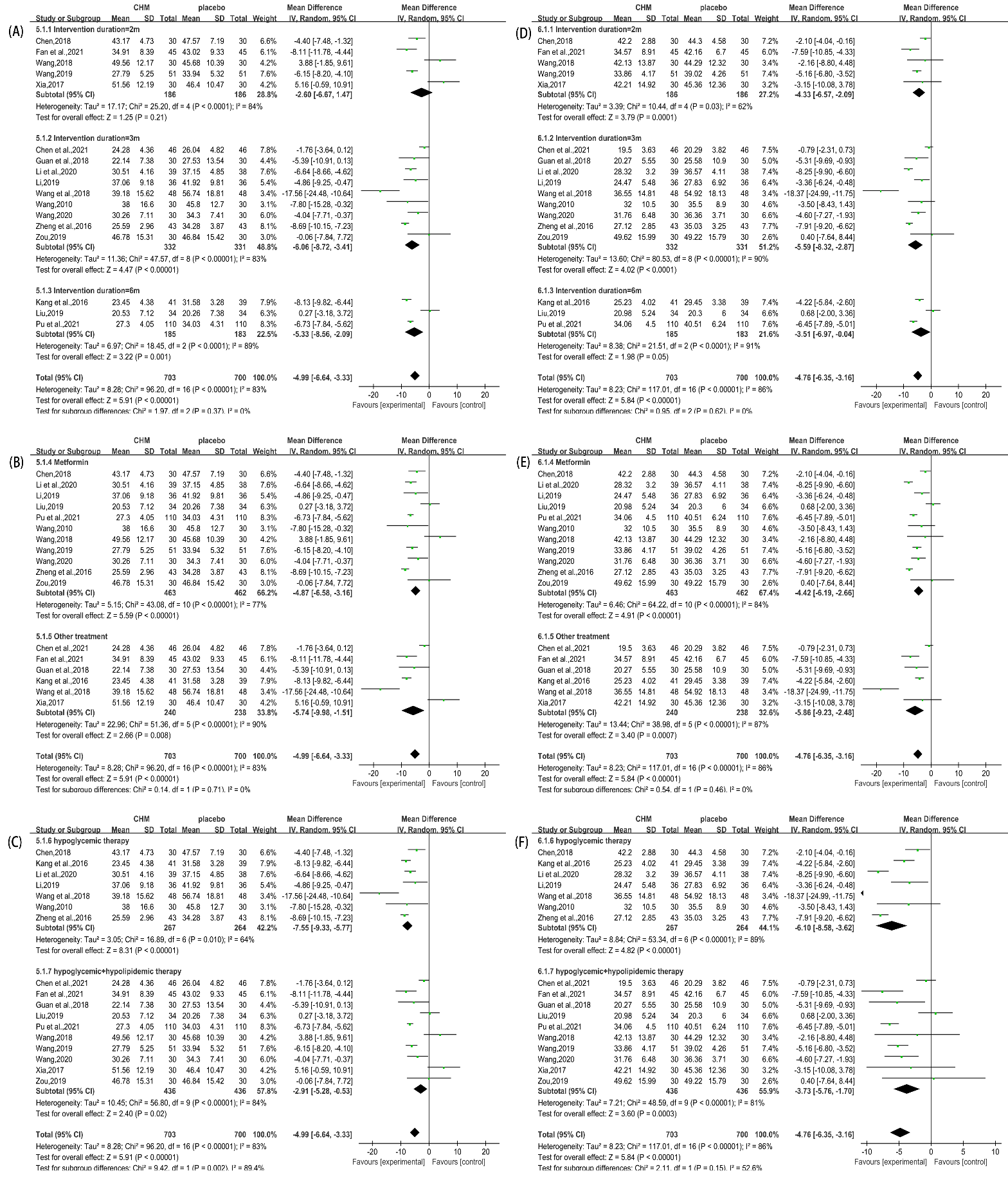


(A)-(C): subgroup analysis for ALT. (D)-(F): subgroup analysis for AST.

4.Subgroup analysis for HOMA-IR and FBG.


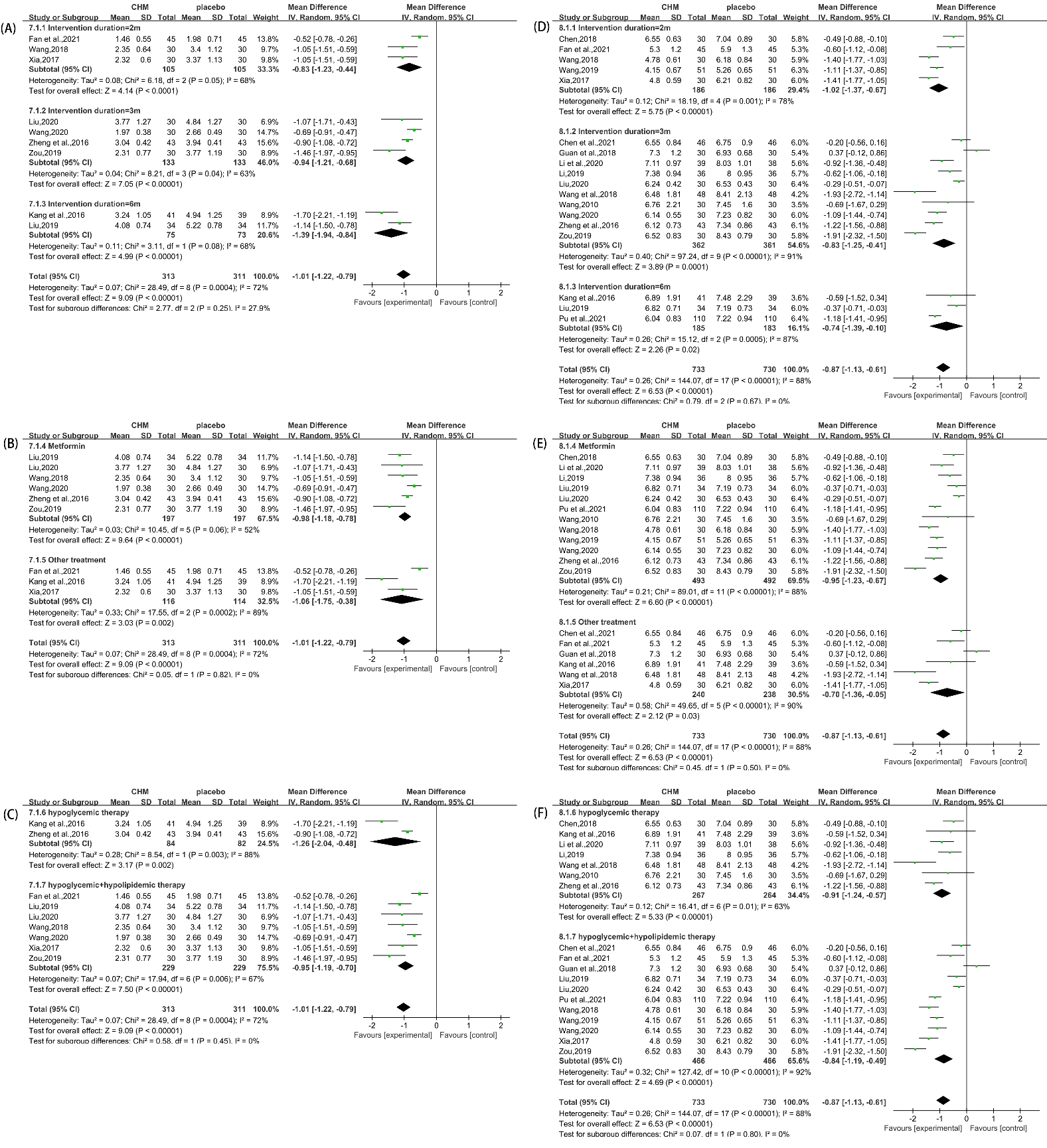


(A)-(C): subgroup analysis for HOMA-IR. (D)-(F): subgroup analysis for FBG.

5.Subgroup analysis for 2hPG and BMI.


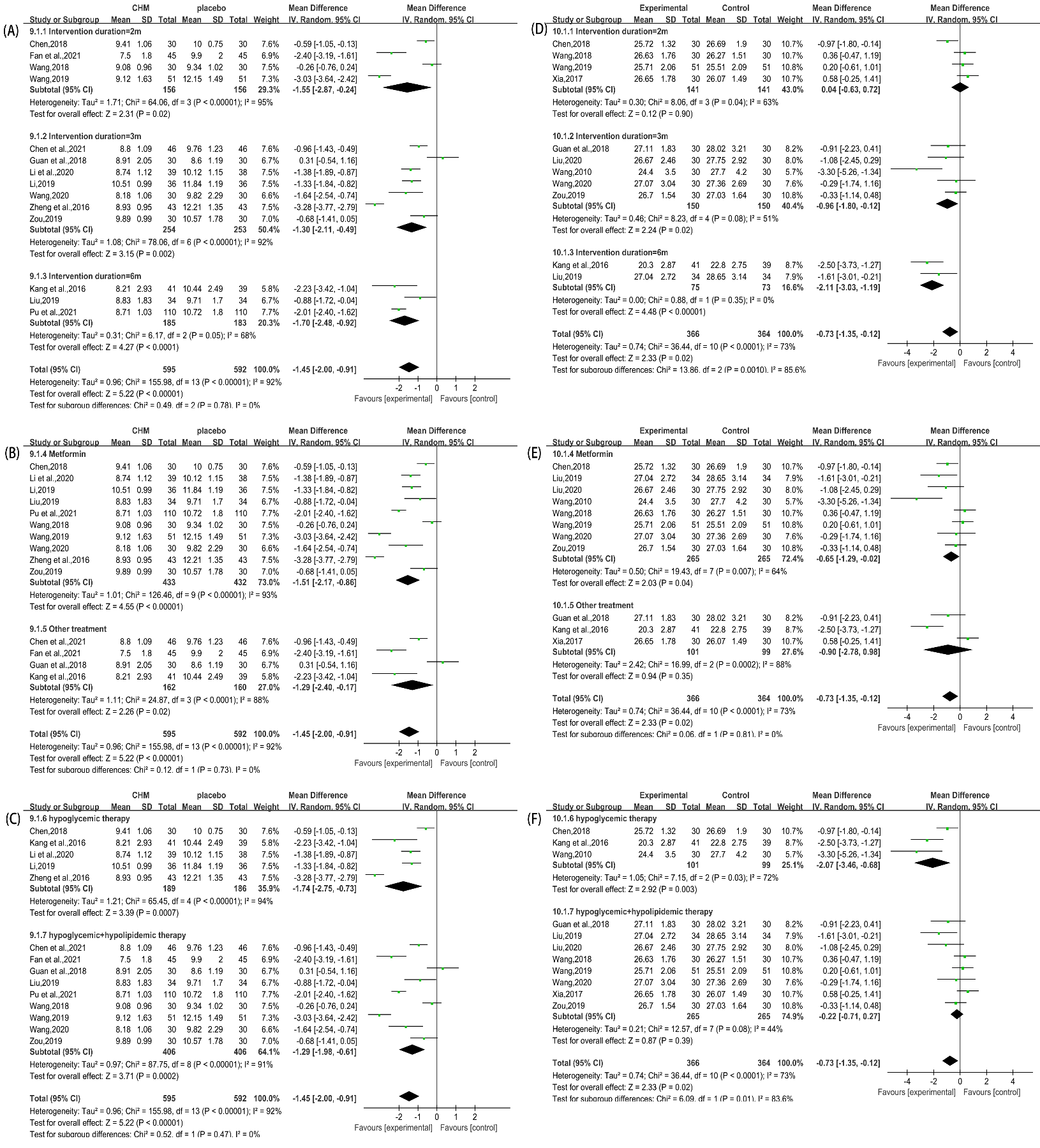


(A)-(C): subgroup analysis for 2hPG. (D)-(F): subgroup analysis for BMI.

# Supplementary Figure S2 Egger’s plot


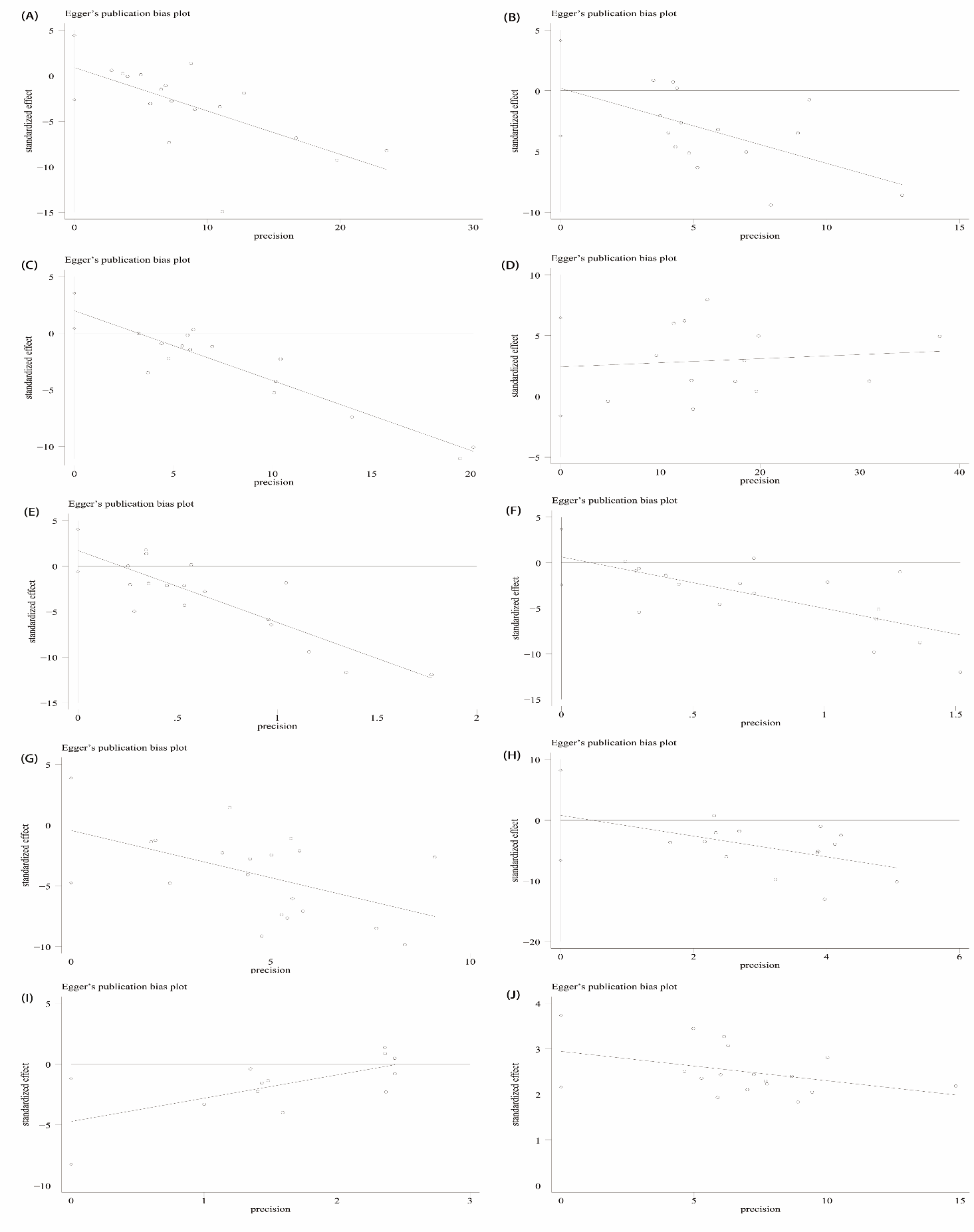


(A) triglyceride; (B) total cholesterol;(C) low-density lipoprotein cholesterol;(D) high-density lipoprotein cholesterol;(E) alanine transaminase;(F) aspartate transaminase;(G) fasting blood glucose;(H) two-hour postprandial glucose;(I) body mass index;(J) overall effective rate.

# Supplementary Figure S3 Sensitivity analysis


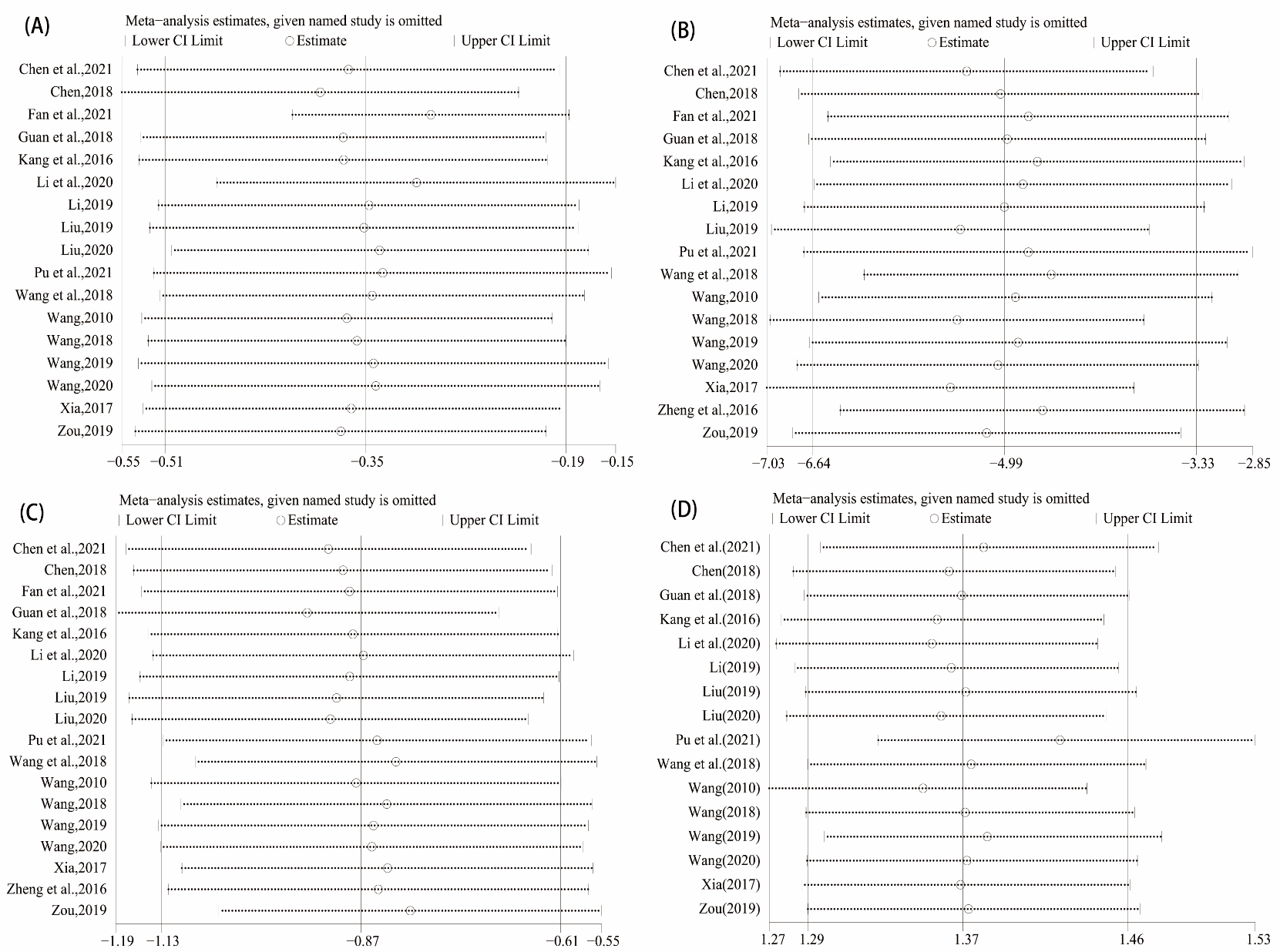


(A) triglyceride; (B) alanine transaminase; (C) fasting blood glucose; (D) overall effective rate.
